# Supplementary material for: Identification of thermotolerant non-canonical PAMs for robust one-pot CRISPR-Cas12a detection
Source: Nat Commun. 2026 Jan 16;17:1771. doi: 10.1038/s41467-026-68476-3 (PMC12917250; doi:10.1038/s41467-026-68476-3)
Supplement: Supplementary file 1 — Supplementary Information [file 41467_2026_68476_MOESM1_ESM.pdf]

Supplementary Information for

Identification of Thermotolerant Non-canonical PAMs for Robust One-Pot CRISPR-Cas12a Detection

Tian Tian[1]†*, Ting Zhang[1]†, Wanting Zhang[1]†, Zhiqiang Qiu[1], Xinyi Guo[1], Yuxin Chen[1], Mei Lin[1], Weiwei Qi[1], Yuting Shen[1], Mengen Hao[1], Hongrui Xiao[1], Bo Xiang[2], Feibiao Pang[3], Jinzhao Song[4]*, Baoqing Sun[2]*, Meng Cheng[2]* and Xiaoming Zhou[1,5]*

1. School of Life Sciences, South China Normal University, Guangzhou, 510631, China.
2. The First Affiliated Hospital of Guangzhou Medical University, Guangzhou, 510120, China.
3. Hangzhou EzDx Technology Co., Ltd., Hangzhou, Zhejiang 311231, China.
4. Hangzhou Institute of Medicine, Chinese Academy of Sciences, Hangzhou, Zhejiang 310022, China.
5. MOE Key laboratory of Laser Life Science & Guangdong Provincial Key Laboratory of Laser Life Science, School of Optoelectronic Science and Engineering, South China Normal University, Guangzhou 510631, China.

† These authors contributed equally.
*To whom correspondence should be addressed.
*Tian Tian
Email: ttian@m.scnu.edu.cn
*Jinzhao Song
Email: songjinzhao@ucas.ac.cn
*Baoqing Sun
Email: sunbaoqing@vip.163.com
*Meng Cheng
Email: ChengMeng@ghmu.edu.cn
*Xiaoming Zhou
Email: zhouxm@scnu.edu.cn
ORCID: 0000-0001-5597-4804

Contents

Supplementary Figure

Supplementary Figure.1 Sequencing of 5 targets from comprehensive PAM target DNA library.

Supplementary Figure.2 Fluorescence kinetics of 256 PAMs at 37℃ and 45℃.

Supplementary Figure.3 Non-canonical PAMs classification at different reaction temperatures.

Supplementary Figure.4 PAM sequences logo for LbCas12a identified using *in vitro* collateral cleavage efficiency.

Supplementary Figure.5 Evaluation of bad-performing non-canonical PAM-mediated collateral activity of Cas12a at different temperatures.

Supplementary Figure.6 Evaluation of the *trans*-cleavage activity of several TTTA-crRNAs at 60℃.

Supplementary Figure.7 Evaluation of the *trans*-cleavage activity of LbCas12a activated by ssDNA target at different temperatures.

Supplementary Figure.8 PS modification contributes to enhance the heat tolerance of crRNAs.

Supplementary Figure.9 Structural comparison of enAsCas12a and LbCas12a-Mut.

Supplementary Figure.10 Validation of the high-temperature *trans*-cleavage activity of LbCas12a-Mut under two buffer conditions.

Supplementary Figure.11 Evaluation the upper temperature limit of *trans*-cleavage activity for two LbCas12a in Tango buffer.

Supplementary Figure.12 Evaluation of the *trans*-cleavage activity of AsCas12a and FnCas12a at different temperatures.

Supplementary Figure.13 Maintenance of the *trans*-cleavage activity of activated LbCas12a at different temperatures.

Supplementary Figure.14 Comparison of two treatments before the electrophoretic analysis of the *cis*-cleavage of Cas12a.

Supplementary Figure.15 Evaluation of dsDNA cleavage by ssDNA target activated Cas12a.

Supplementary Figure.16 Detection sensitivity of two CRISPR-based one-pot methods.

Supplementary Figure.17 Evaluation of the sensitivity of POP-CRISPR and sPAMC on RNA target.

Supplementary Figure.18 The accumulation of RAA amplicons in the POP-CRISPR system.

Supplementary Figure.19 Reproducibility of POP-CRISPR platform.

Supplementary Figure.20 Schematic illustration of the synergistic regulation of Cas12a detection specificity by temperature and non-canonical PAMs.

Supplementary Figure.21 Optimization of extraction-free rapid lysis protocol.

Supplementary Figure.22 Stability assessment of rapid lysis release nucleic acid samples.

Supplementary Figure.23 Overall design of the mini-device.

Supplementary Figure.24 The operation workflow of on-site MP detection by POP-CRISPR using mini-device.

Supplementary Figure.25 Real-time fluorescence curves obtained from POP-CRISPR experiments

performed on Bio-Rad CFX96 Touch Real-Time PCR System.

Supplementary Table

Supplementary Table 1 Nucleic acid sequences used in **Figure 1.**

Supplementary Table 2 Nucleic acid sequences used in **Figure 2 and Figure 3.**

Supplementary Table 3 Nucleic acid sequences used in **Figure 4.**

Supplementary Table 4 Nucleic acid sequences used in **Figure 5 and Figure 6.**

Supplementary Table 5 Plasmid sequences used in this study (ASFV).

Supplementary Table 6 Plasmid sequences used in this study (SARS-CoV-2 S-gene).

Supplementary Table 7 Plasmid sequences used in this study (SARS-CoV-2 N-gene).

Supplementary Table 8 The *Ct* values of 33 HPV-16 clinical samples measured by qRT-PCR.

Supplementary Table 9 The *Ct* values of 67 clinical MP samples measured by qRT-PCR.

Supplementary Table 10 The *Ct* values of 11 clinical MP samples for macrolide-resistant detection measured by qRT-PCR.

Supplementary Table 11 The *Ct* values of 27 clinical MP samples used in Fig.6c-d measured by qRT-PCR.

Supplementary Table 12 Comparison between POP-CRISPR and qPCR.

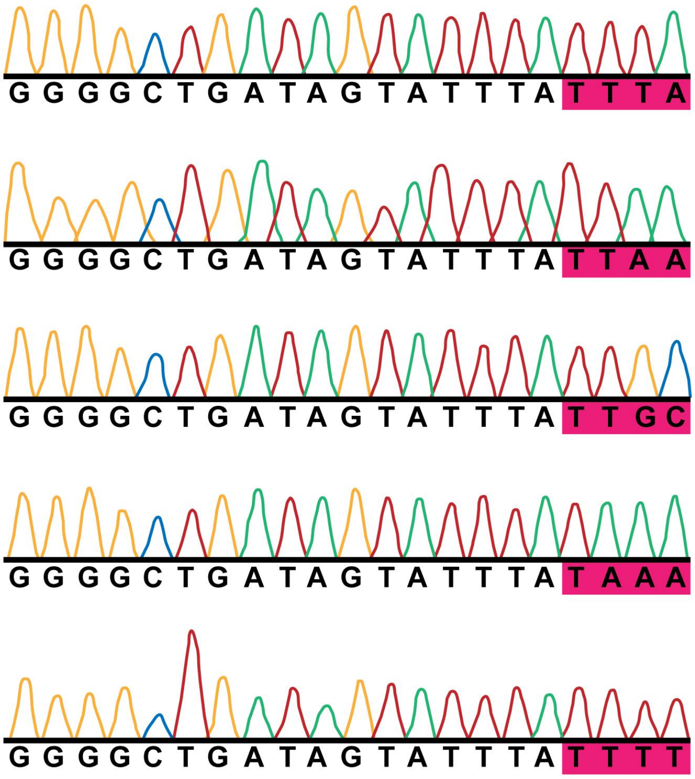

Supplementary Figure 1 | Sequencing of 5 targets from comprehensive PAM target DNA library. The figure shows representative Sanger sequencing results confirming the correct insertion of the five selected PAM target sequences. These sequencing results validate the integrity and accuracy of the constructed PAM target DNA library used for downstream assays.

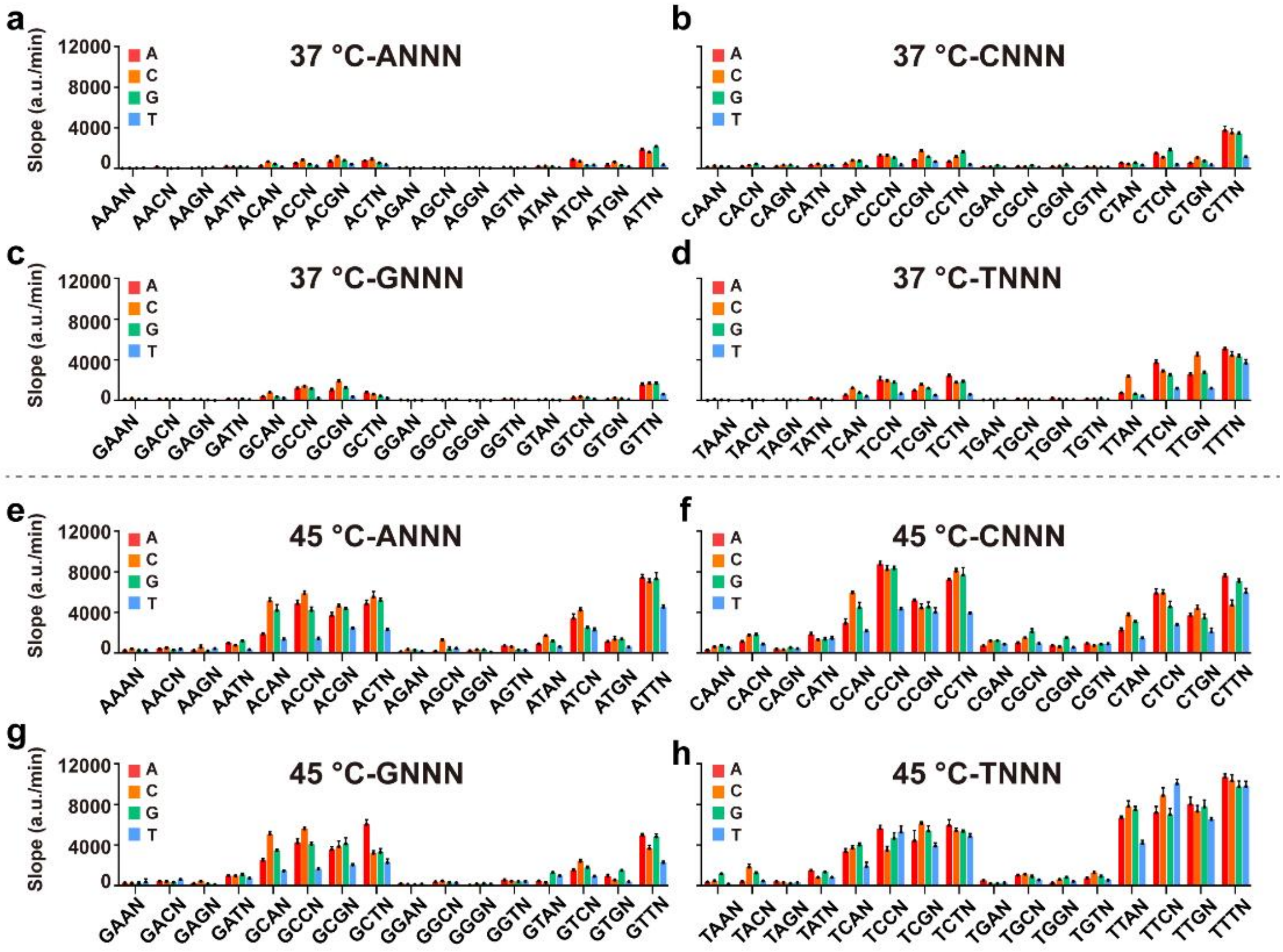

Supplementary Figure 2 | Fluorescence kinetics of 256 PAMs at 37℃ and 45℃. a-d Fluorescence kinetics of 256 PAMs were measured in collateral activity experiments at 37℃. e-h Fluorescence kinetics of 256 PAMs were measured in collateral activity experiments at 45℃. The slope represents the rate of fluorescence change during the first four minutes, prior to reaching the maximum fluorescence value. The concentrations of dsDNA targets were 100 pM. Data are represented as mean ± standard error (n=3 technical replicates).

	Best-performing PAM	Mediocre-performing PAM	Bad-performing PAM
37 °C	CTTV TTCA TTGC (5)	ATRC RTTV CCAC CCKS CTCV CTTT BCCV GCGS TCAC TCKV TTAV TTCB TTGD (45)	NANN RTTT SCCY NGNN RTVN YCAD ACYN ACRD TCBT SCGW CTCT CTRN GCWN (202)
45 °C	WCAC ACCM ACGS ACTV ATCC MTTN CCAV CCBN CTAC CTCN CTGV GCAS GCYV GCGN GTTV TCBN TTVN (82)	AATG ACAD ACCK ACGW ACTT AGCC ATAS ATCD ATGV CACV CATN CCAT CGAG CGCS CGGG CGTC CTAD CTGT GCAW GCYT GTAK GTCN GTGR GTTT TAAG TACC TATR TCAT TGCV TGTS (58)	AAGN AATH AGDN AGCD ATAW ATGT CARN CACT CGRH CGCW CGTD GRNN GTAM GTGY TAAH TACD TAGN TATY TGRN TGCT TGTW (112)

R=A/G; Y=C/T; M=A/C; K=G/T; S=G/C; W=A/T; H=A/T/C; B=G/T/C; V=G/A/C; D=G/A/T; N=A/T/C/G

Supplementary Figure 3 | Non-canonical PAMs classification at different reaction temperatures. We counted the time (t) to reach half of the maximum fluorescence value at a detection target concentration of 1 nM at each PAM site and used this criterion to classify all 252 non-classical PAMs into three categories: (1) Best-performing PAM ($t \leq 5$ min), (2) Mediocre-performing PAM ($5 \text{ min} < t \leq 10$ min), (3) Bad-performing PAM ($t > 10$ min).

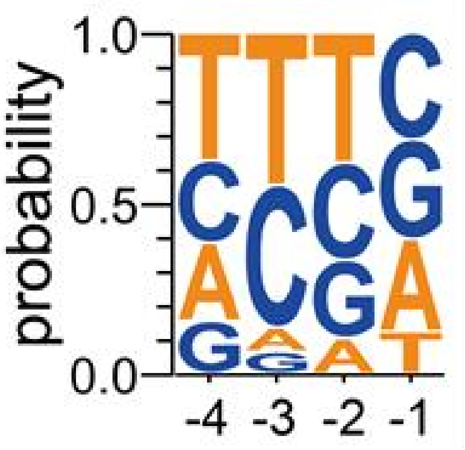

Supplementary Figure 4 | PAM sequences Logo for LbCas12a identified using *in vitro* collateral cleavage efficiency. To obtain Sequence Logo plots, the different PAM-mediated fluorescence rates of the *trans*-cleavage were first converted into FASTA files and then imported into WebLogo 3 (https://weblogo.threeplusone.com/create.cgi) for plotting.

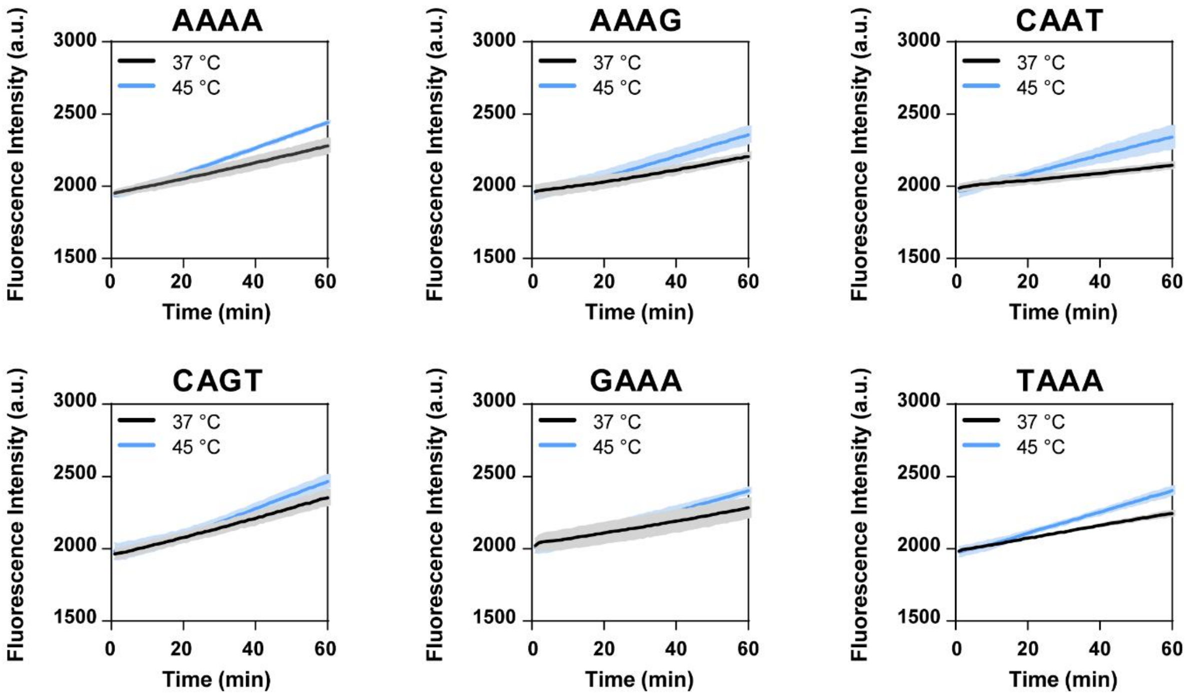

Supplementary Figure 5 | Evaluation of bad-performing non-canonical PAM-mediated collateral activity of Cas12a at different temperatures. 6 crRNAs based on bad-performing PAM including AAAA, AAAG, CAAT, CAGT, GAAA and TAAA were designed to target S gene for collateral activity evaluation at 37 and 45 ℃. Data are represented as mean ± standard error (n=3 technical replicates).

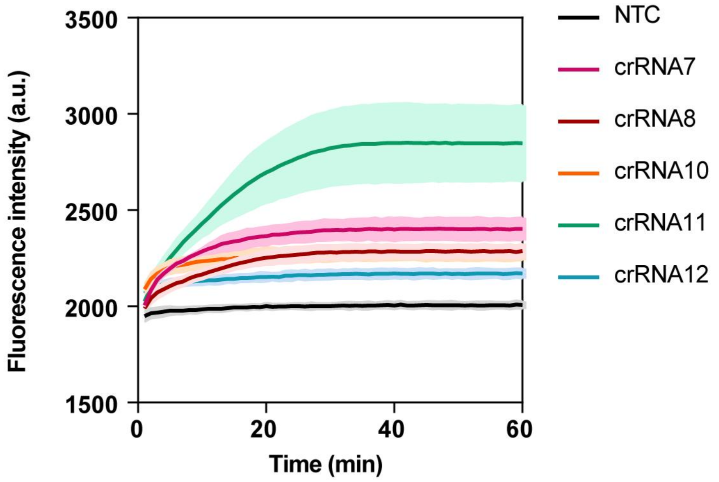

Supplementary Figure 6 | Evaluation of the *trans*-cleavage activity of several TTTA-crRNAs at 60°C. These crRNAs are from **Fig.2c**, which demonstrated sustained *trans*-cleavage activity at 57°C. Data are represented as mean ± standard error (n=3 technical replicates).

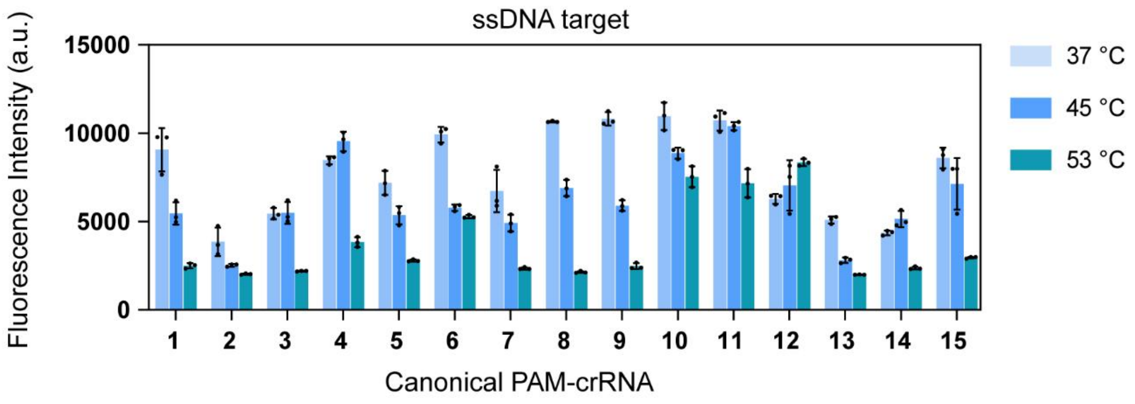

Supplementary Figure 7 | Evluation of the *trans*-cleavage activity of LbCas12a activated by ssDNA target at different temperatures. 15 crRNAs are from **Fig.2c** and a final concentration of 100pM of ssDNA targets was used in the experiment. Values are shown as endpoint fluorescence at 60 minutes. Data are represented as mean ± standard error (n=3 technical replicates).

crRNA2-8PS	G*U*A*A*UUUCUAAGUGUAGAUCAUUCAACUCAGGACU*U*G*U*U
crRNA5-8PS	G*U*A*A*UUUCUAAGUGUAGAUAGAAUAUUGAUGGUUA*U*U*U*U
crRNA13-8PS	G*U*A*A*UUUCUAAGUGUAGAUGAACCAUUGGUAGAUU*U*G*C*C
crRNA-CTCA-8PS	G*U*A*A*UUUCUAAGUGUAGAUGCCUUUUCUUAUGGAC*C*U*U*G

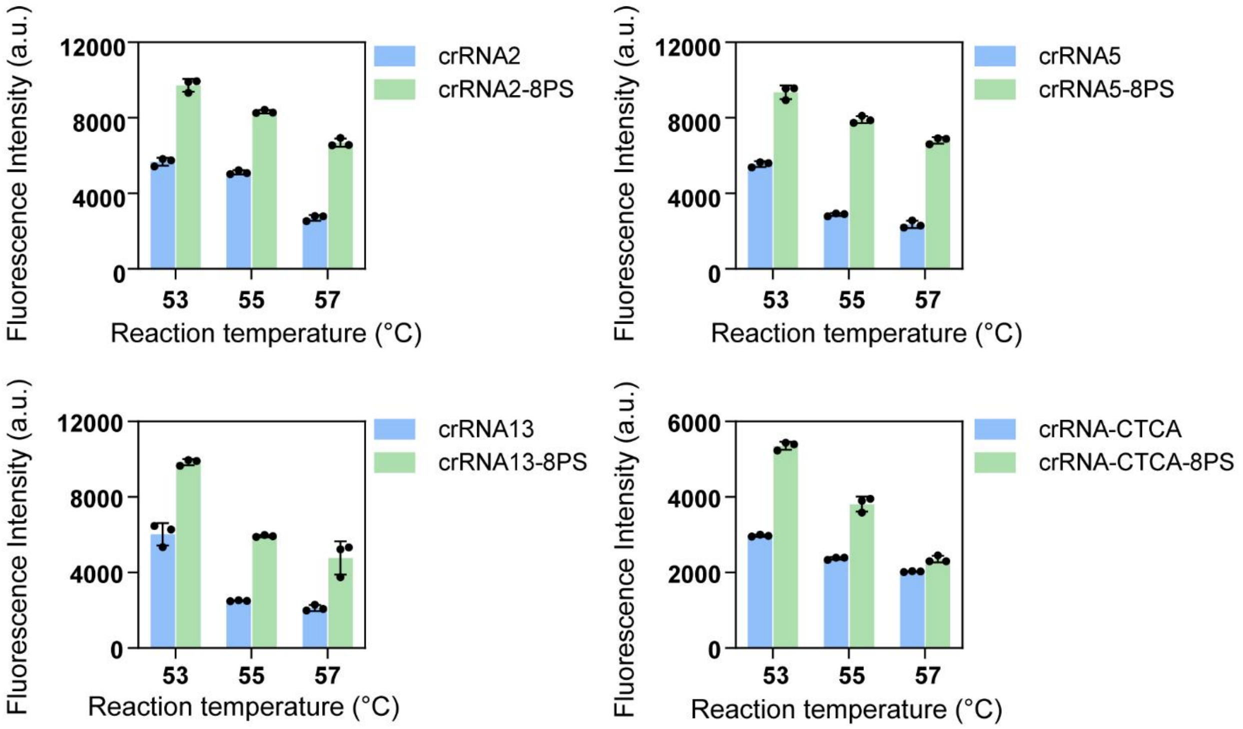

Supplementary Figure 8 | PS modification contributes to enhance the reactivity of crRNAs. Top, PS-modified crRNA sequences. PS modifications are indicated by red asterisks. Bottom, comparison of the *trans*-cleavage activity of unmodified and PS-modified crRNAs at different temperatures (53℃, 55℃, 57℃). Values are shown as endpoint fluorescence at 60 minutes. Data are represented as mean ± standard error (n=3 technical replicates).

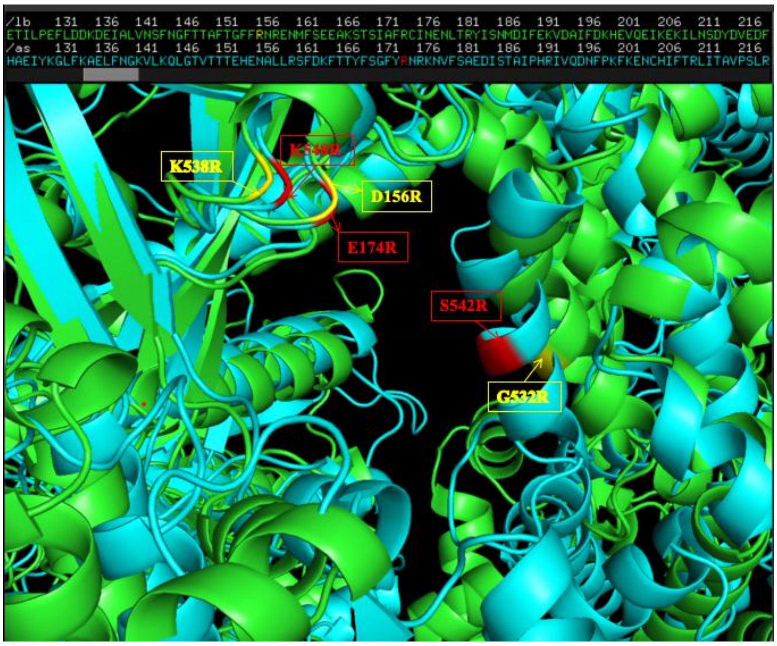

Supplementary Figure 9 | Structural comparison of enAsCas12a and LbCas12a-Mut. Predicted three-dimensional structural models of enAsCas12a (blue) and LbCas12a-Mut (green) were generated using AlphaFold3 (https://alphafoldserver.com/) and visualized with PyMOL (https://www.pymol.org/). Structural alignment revealed that the mutation sites—highlighted in red for enAsCas12a and in yellow for LbCas12a-Mut—are spatially well conserved and located within the same functional domain, suggesting that LbCas12a-Mut is likely to possess a similar functional mechanism to enAsCas12a.

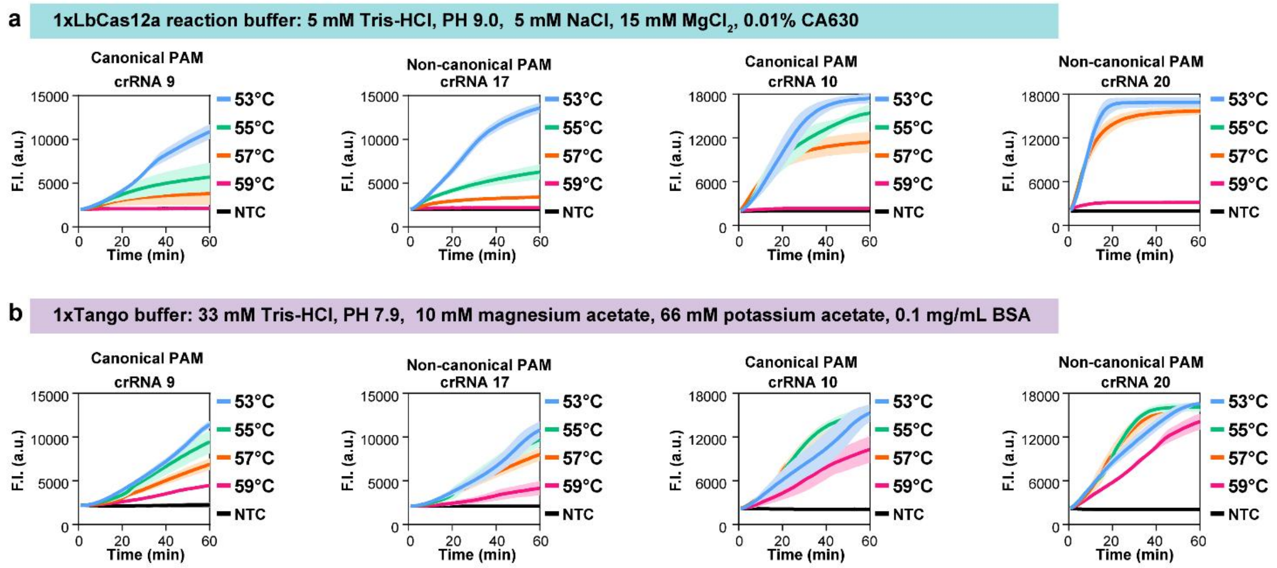

Supplementary Figure 10 | Validation of the high-temperature *trans*-cleavage activity of LbCas12a-Mut under two buffer conditions. a Real-time fluorescence kinetics of four crRNAs—including two heat-sensitive crRNAs (crRNA9 and crRNA17) and two heat-tolerant crRNAs (crRNA10 and crRNA20)—in LbCas12a buffer. **b** Real-time fluorescence kinetics of the same crRNAs in Tango buffer. The target used was the PCR-amplified S-gene fragment at a final concentration of 100 pM. Data are represented as mean ± standard error (n=3 technical replicates).

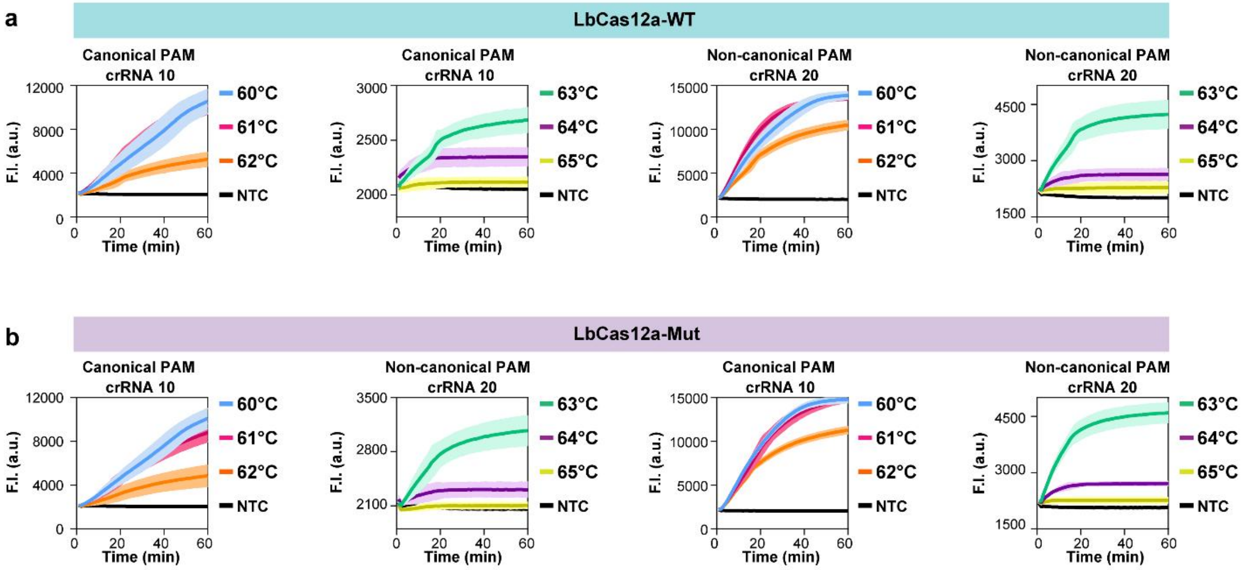

Supplementary Figure 11 | Evaluation the upper temperature limit of *trans*-cleavage activity for two LbCas12a in Tango buffer. a Real-time fluorescence kinetics of LbCas12a-WT guided by two thermotolerant crRNAs (crRNA10 and crRNA20) in Tango buffer at temperatures from 60 to 65 °C. **b** Real-time fluorescence kinetics of LbCas12a-Mut guided by two thermotolerant crRNAs (crRNA10 and crRNA20) in Tango buffer at temperatures from 60 to 65 °C. The target used was the PCR-amplified S-gene fragment at a final concentration of 100 pM. Data are represented as mean ± standard error (n=3 technical replicates).

a

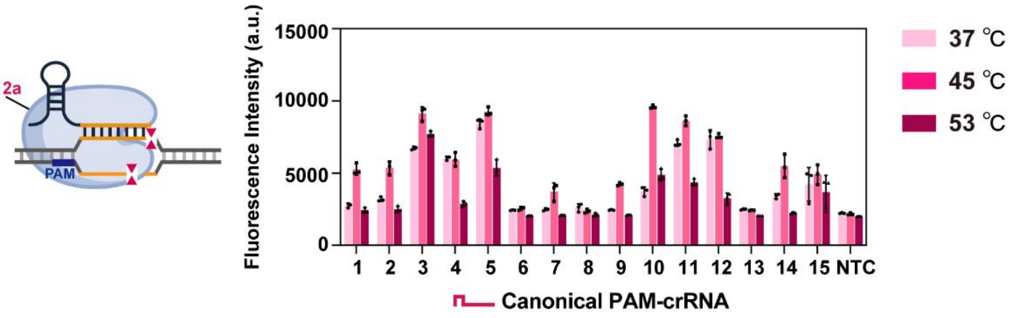

b

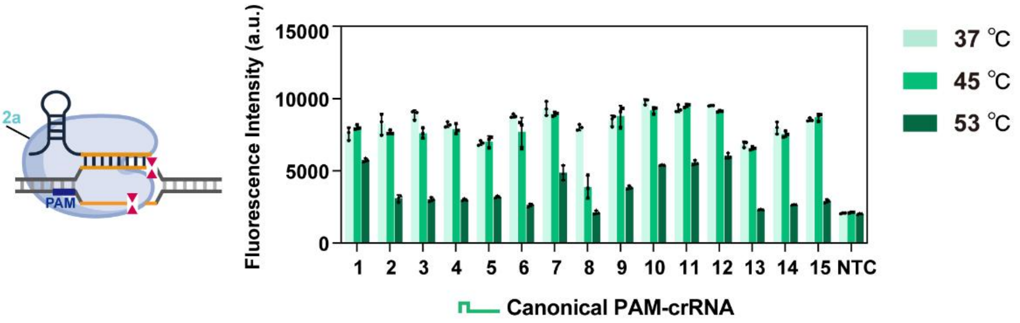

Supplementary Figure 12 | Evaluation of the *trans*-cleavage activity of activated AsCas12a and FnCas12a at different temperature conditions. a Trans-cleavage activity of activated AsCas12a at different temperatures. **b** Trans-cleavage activity of activated FnCas12a at different temperatures. 15 crRNAs are from **Fig.2c** and a final concentration of 100pM of dsDNA target was used in the experiment. Values are shown as endpoint fluorescence at 60 minutes. Data are represented as mean ± standard error (n=3 technical replicates).

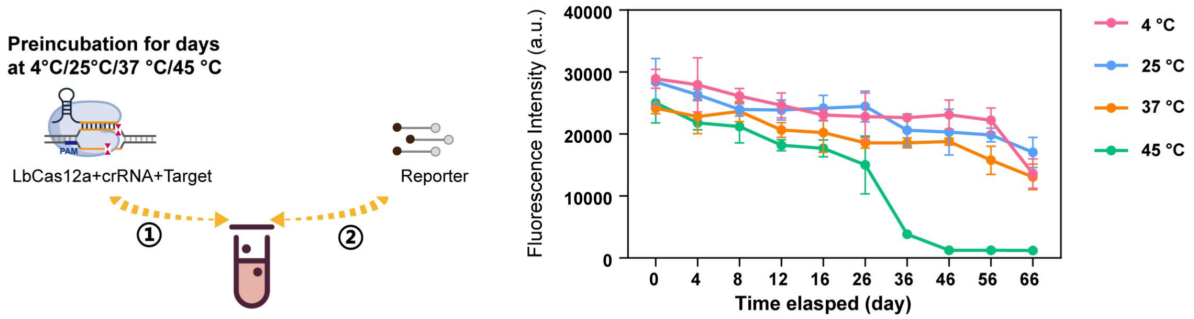

Supplementary Figure 13 | Maintenance of the *trans*-cleavage activity of activated LbCas12a at different temperatures. Left, reaction system preparation. Cas12a reaction components except the reporter were prepared and aliquoted, and subsequently incubated at 4°C/25°C/37°C/45°C. One aliquot was taken every 4 days for the fist 16 days, added to the reporter, allowed to react and fluorescence collected at 45°C, and tested every 10 days thereafter. Right, line plots showing how activated Cas12a retains *trans*-cleavage activity at different temperatures. Values are shown as endpoint fluorescence at 60 minutes. Data are represented as mean ± standard error (n=3 technical replicates).

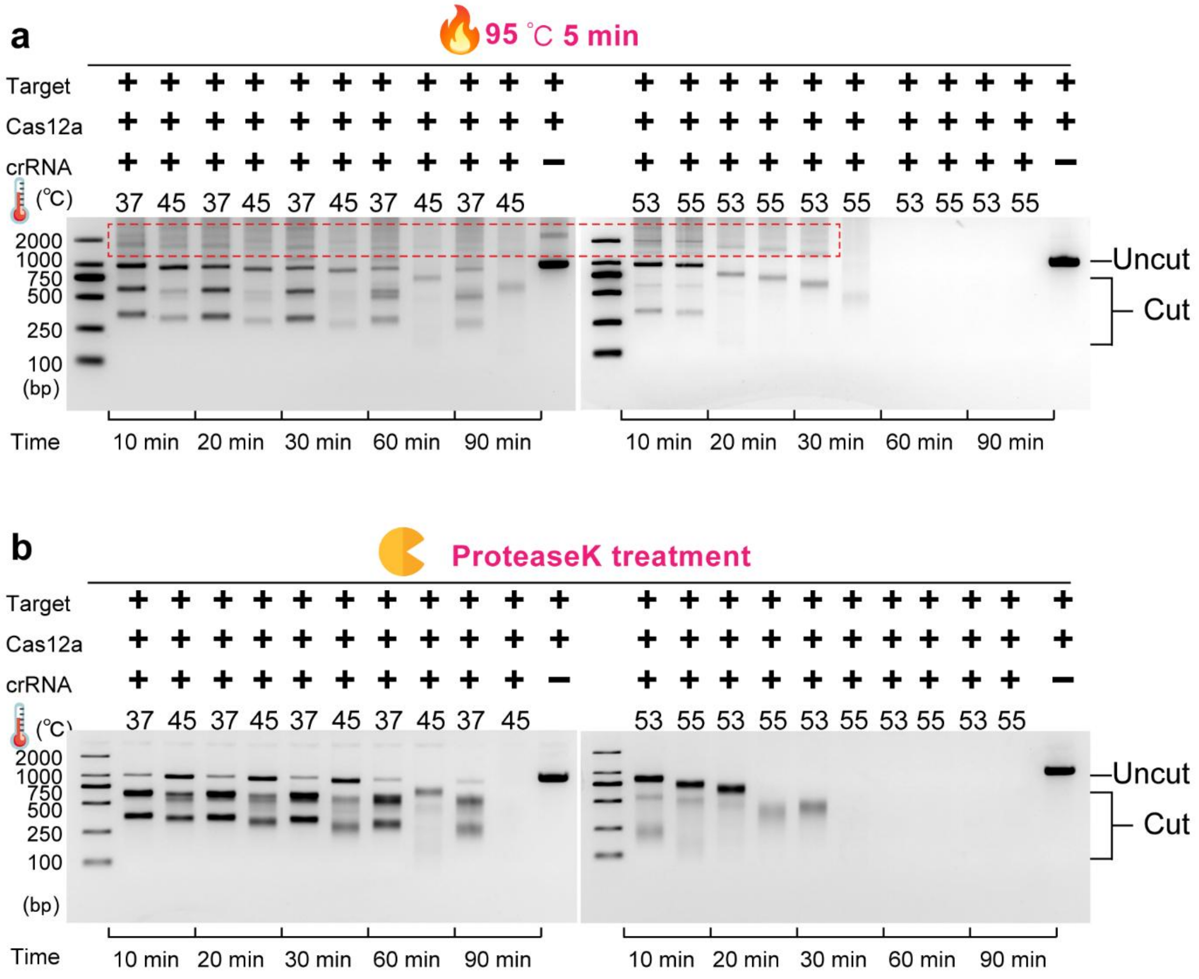

Supplementary Figure 14 | Comparison of two treatments before the electrophoretic analysis of the *cis*-cleavage of Cas12a. a High temperature treatment. Prior to electrophoretic analysis, the samples were treated with 95℃ for 5 minutes. The area framed by the red dashed line shows the non-specific binding bands of Cas12a and dsDNA. **b** Protease K treatment. Prior to electrophoretic analysis, 2 μL protease K was added and treated with 37℃ for 15 minutes.

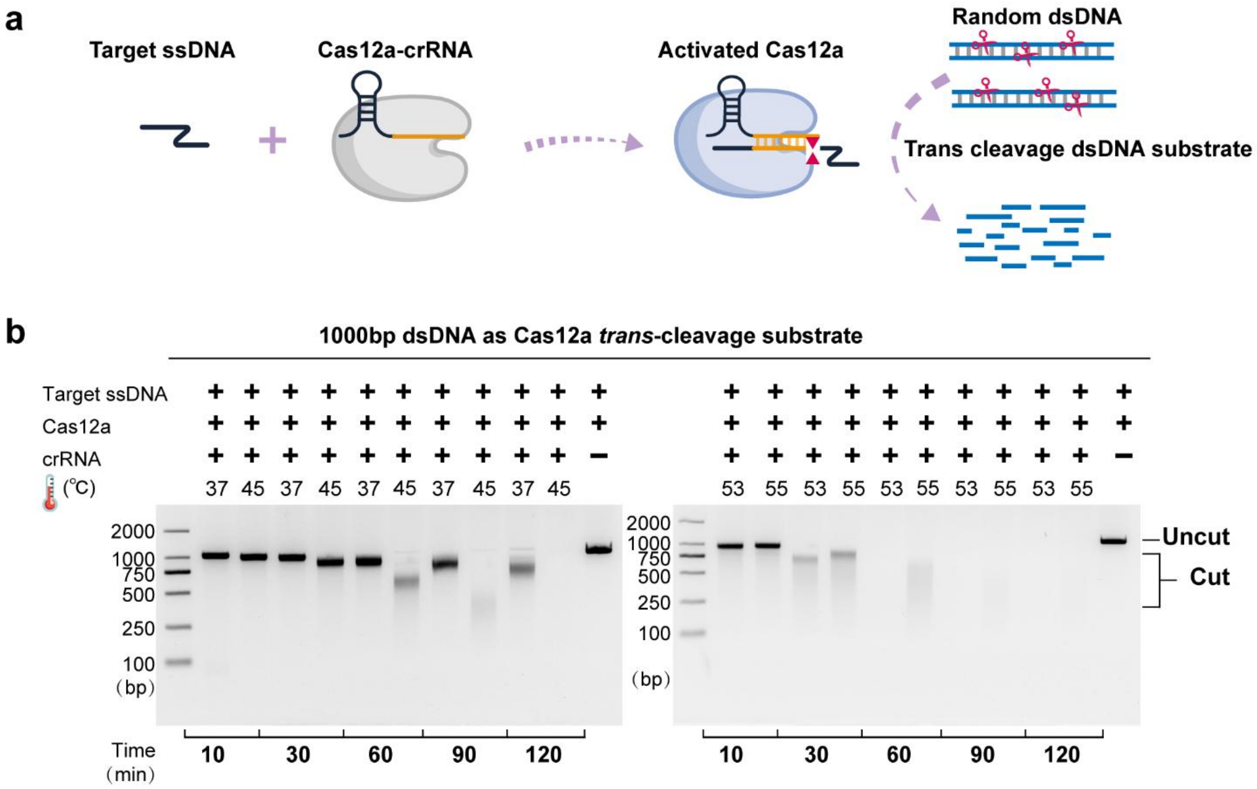

Supplementary Figure 15 ┃ Evaluation of dsDNA cleavage by ssDNA target activated Cas12a. a Schematic of *trans*-degradation of dsDNA substrates by ssDNA-activated Cas12a. **b** Analysis of *trans*-degradation of dsDNA substrates by ssDNA-activated Cas12a at different temperatures. *Trans*-degradation activities were analysed using 2% agarose gels at 10, 30, 60, 90 and 120 minutes across different temperatures (37-55°C). The final concentration of dsDNA used was 120 nM.

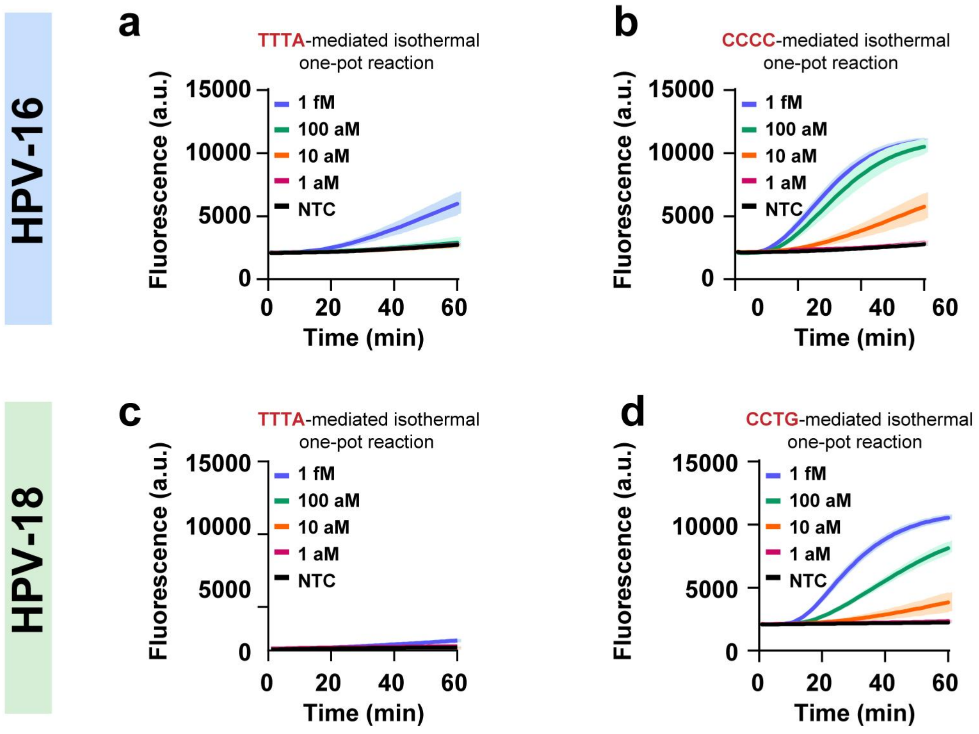

Supplementary Figure 16 | Detection sensitivity of two CRISPR-based one-pot methods. a Real-time fluorescence curves were tested by TTTA-mediated isothermal one-pot reaction for detection of HPV-16 DNA at the concentrations of 1000, 100, 10, 1, and 0 aM. **b** Real-time fluorescence curves were tested by sPAMC for detection of HPV-16 DNA at the concentrations of 1000, 100, 10, 1, and 0 aM. **c** Real-time fluorescence curves were tested by TTTA-mediated isothermal one-pot reaction for detection of HPV-18 DNA at the concentrations of 1000, 100, 10, 1, and 0 aM. **d** Real-time fluorescence curves were tested by sPAMC for detection of HPV-18 DNA at the concentrations of 1000, 100, 10, 1, and 0 aM. Data are represented as mean ± standard error (n=3 technical replicates).

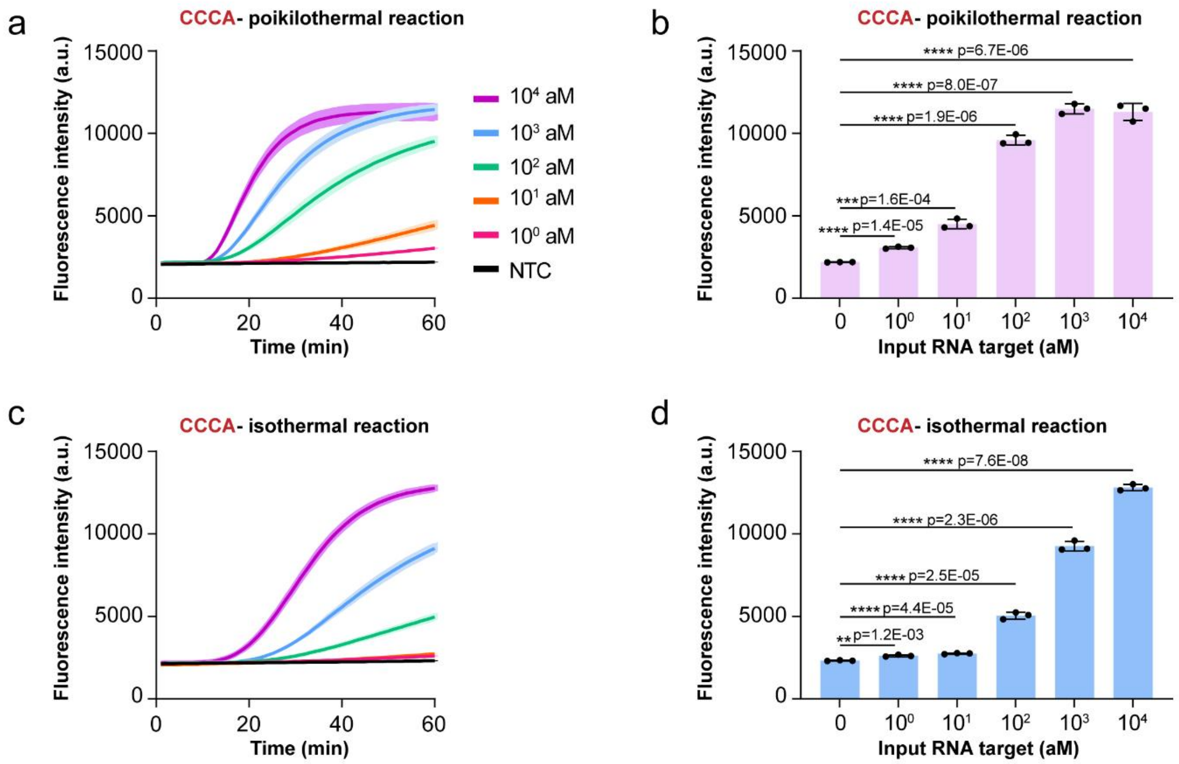

Supplementary Figure 17 | Evaluation of the sensitivity of POP-CRISPR and sPAMC on RNA target. a Real-time fluorescence signal curves for the detection of SARS-CoV-2 N gene by POP-CRISPR. **b** Endpoint fluorescence signals (60 min) for each RNA target concentration in panel **a**. **c** Real-time fluorescence signal curves for the detection of SARS-CoV-2 N gene by sPAMC. d Endpoint fluorescence signals (60 min) for each RNA target concentration in panel **c.** RNA targets were serially diluted with final concentrations of 10 fM, 1 fM, 100 aM, 10 aM, and 1 aM. All experiments were performed in triplicate, with error bars representing the mean values +/- SD (n = 3), and statistical analysis was conducted using a two-tailed t-test. Statistical significance is indicated as follows: n.s. = no significance with P > 0.05, and the asterisks (* P < 0.05; ** P < 0.01; *** P < 0.001; **** P < 0.0001.). a.u. represents arbitrary units.

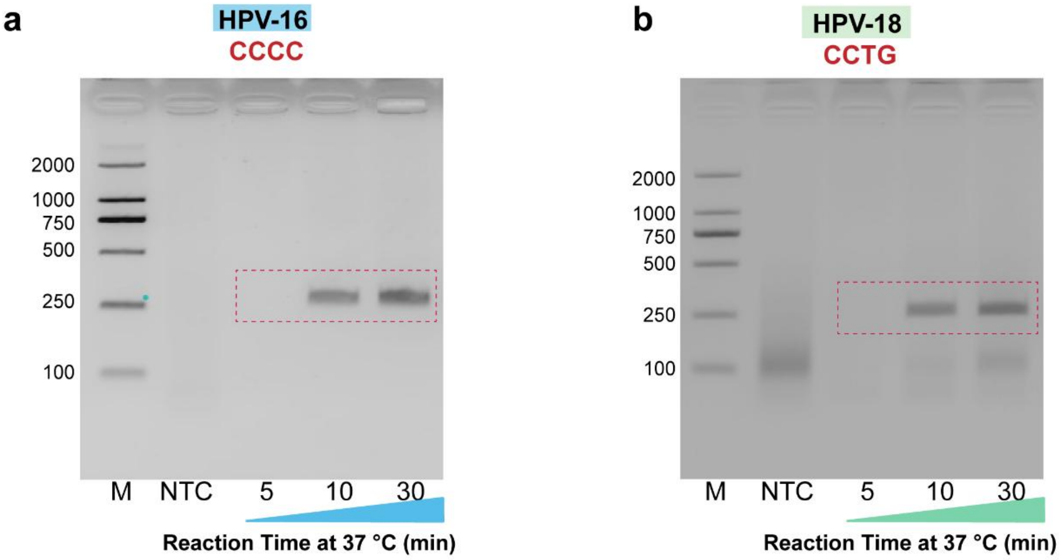

Supplementary Figure 18 The accumulation of RAA amplicons in the POP-CRISPR system. a Reaction mixtures containing 10 nM Cas12a/crRNA RNP and 10 aM HPV-16 dsDNA substrates were incubated at 37 °C for 5, 10, and 30 min. **b** Reaction mixtures containing 10 nM Cas12a/crRNA RNP and 10 aM HPV-16 dsDNA substrates were incubated at 37 °C for 5, 10, and 30 min. The resulting RAA amplicons from both reactions were analyzed in 2% agarose gels.

a

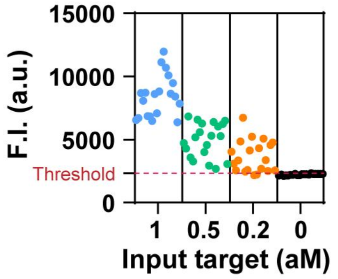

b

Input target (aM)	Input target (copies/reaction)	Positive/tested
1	9	20/20
0.5	4.5	20/20
0.2	1.8	18/20
0	0	0/20

Supplementary Figure 19 | Reproducibility of POP-CRISPR platform. a 20 experiments were repeated independently with HPV-16 DNA detection at a concentration of 9, 4.5, 1.8, and 0 copies/reaction. Threshold was determined by the average fluorescence values of 20 NTC plus 3 times of standard deviation. **b** Positive detection rates of detecting with different concentrations of HPV-16 DNA in (**a**). The volume of the reaction system was 15 μL.

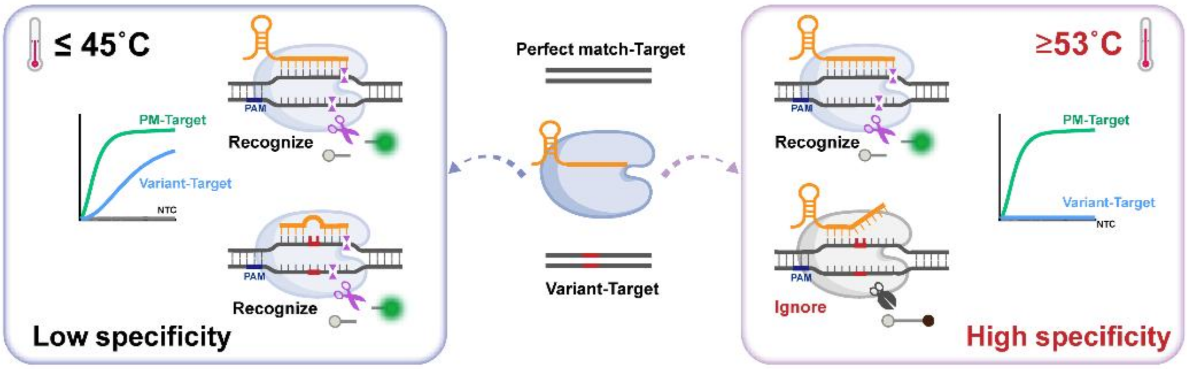

Supplementary Figure 20 | Schematic illustration of the synergistic regulation of Cas12a detection specificity by temperature and non-canonical PAMs. At low temperatures (<45 °C), both perfectly matched and mismatched targets can activate Cas12a due to stable RNP binding. Increasing the temperature (>53 °C) destabilizes mismatched crRNA–DNA pairs, and the weaker affinity of non-canonical PAMs further limits Cas12a activation to fully matched targets, enabling single-base discrimination.

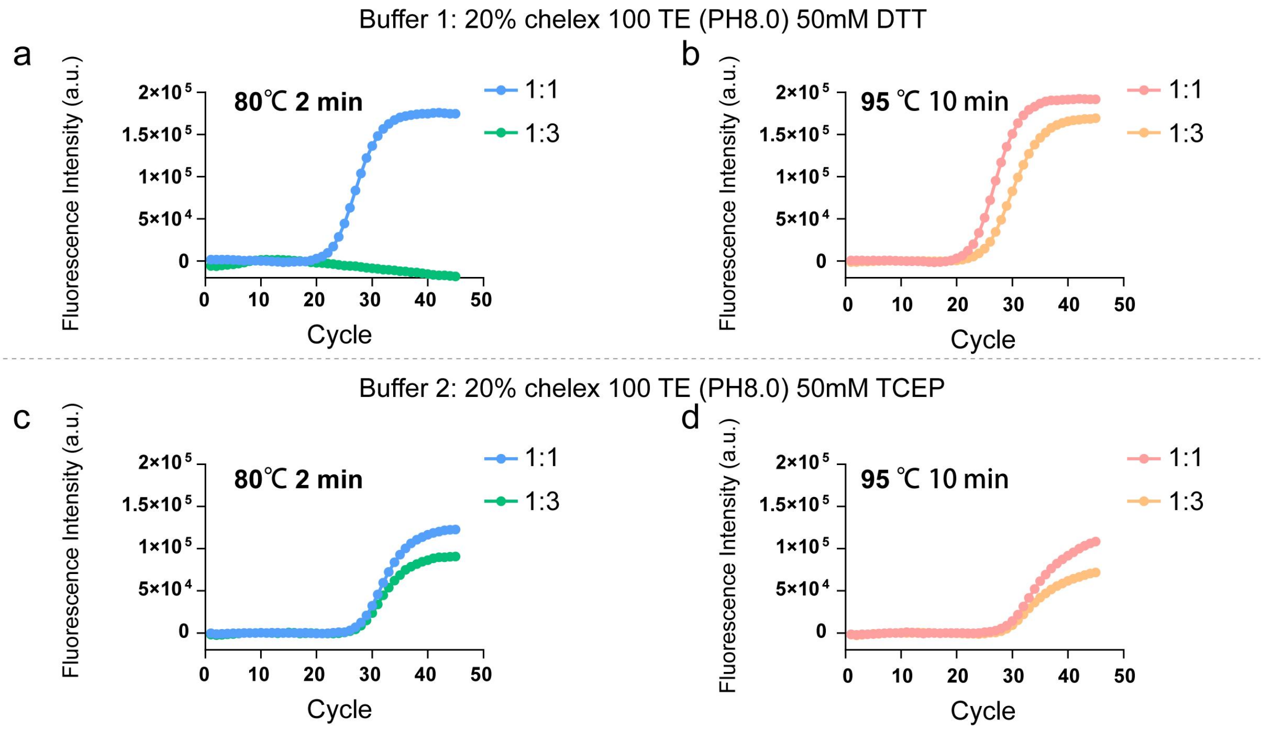

Supplementary Figure 21 | Optimization of extraction-free rapid lysis protocol. a The real-time fluorescence curves of qPCR using lysed swab sample with different ratios of sample to lysis buffer1 (1:1 and 1:3), Lysis condition: 80°C for 2 min. **b** The real-time fluorescence curves of qPCR using lysed swab sample with different ratios of sample to lysis buffer1 (1:1 and 1:3), Lysis condition: 95°C for 10 min. **c** The real-time fluorescence curves of qPCR using lysed swab sample with different ratios of sample to lysis buffer2 (1:1 and 1:3), Lysis condition: 80°C for 2 min. **d** The real-time fluorescence curves of qPCR using lysed swab sample with different ratios of sample to lysis buffer2 (1:1 and 1:3), Lysis condition: 95°C for 10 min.

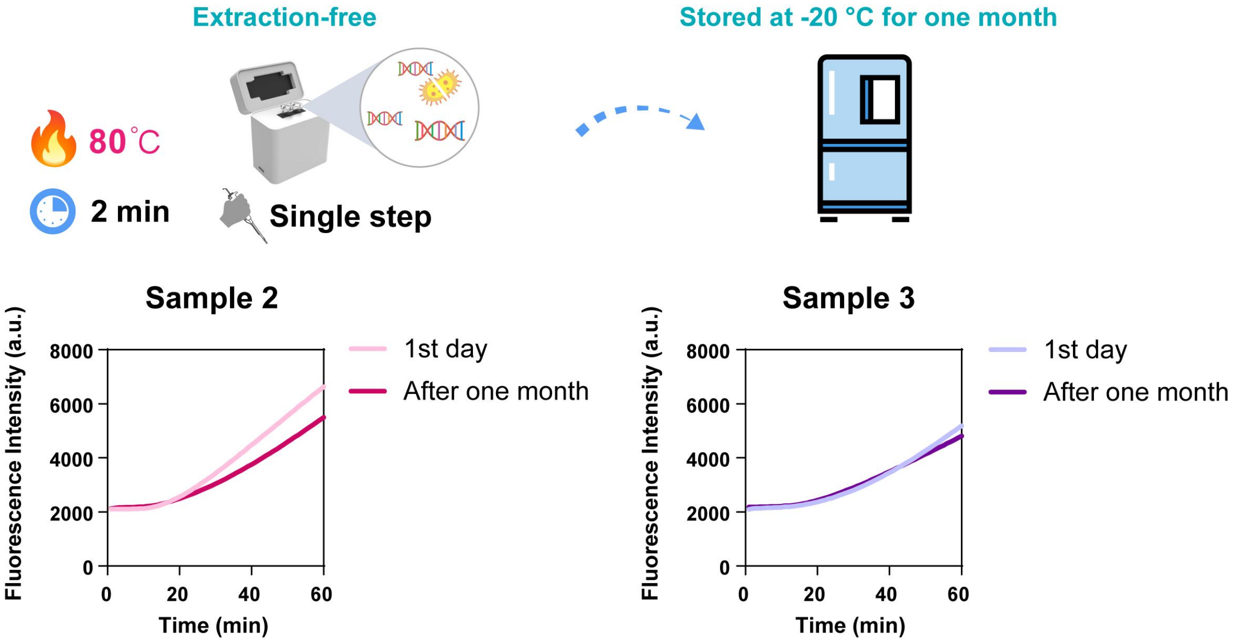

Supplementary Figure 22 | Stability assessment of rapid lysis release nucleic acid samples. Top, schematic of rapid lysis to release nucleic acids and cryogenic storage. Bottom, the real-time fluorescence curves of POP-CRISPR using two lysed samples (sample2 and sample3) at different times of storage.

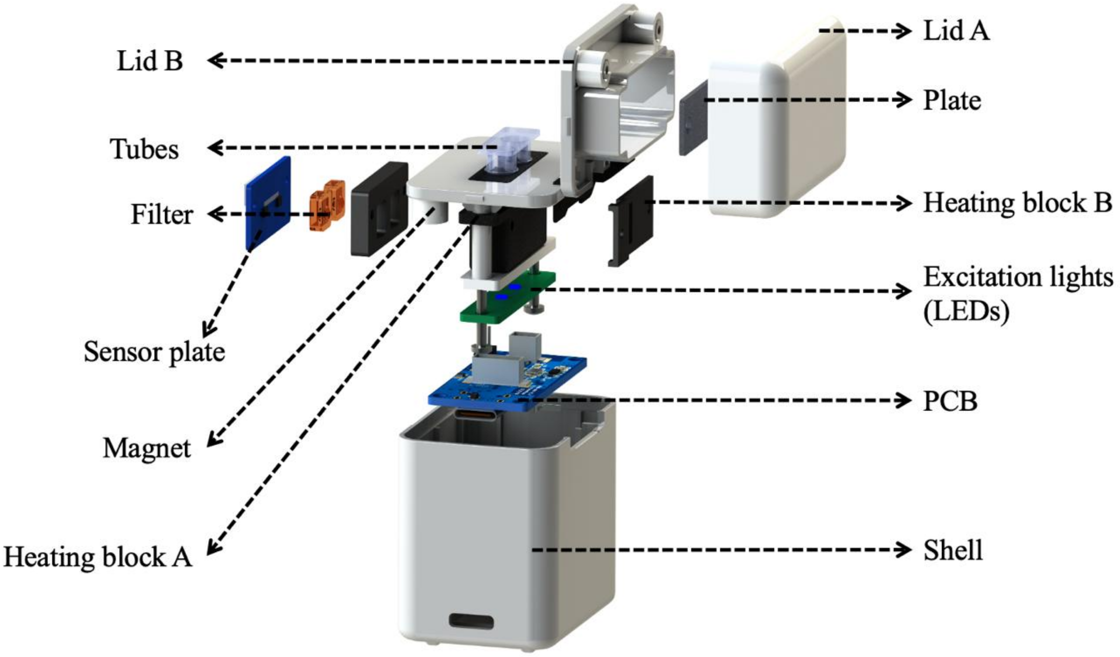

Supplementary Figure 23 | Overall design of the mini-device. The 3D module exploded view of the device, which is composed of a control circuit, a heating module, an excitation light module, a fluorescence sensor module and a heating block. The core detection assembly consists of several key components, including the sensor plate, light filters, filter mounting plate, reaction bearing block, heating sheet, excitation light mounting plate, excitation light (LED) plates, and lower shell mounting plates with magnet mounting positions at both ends.

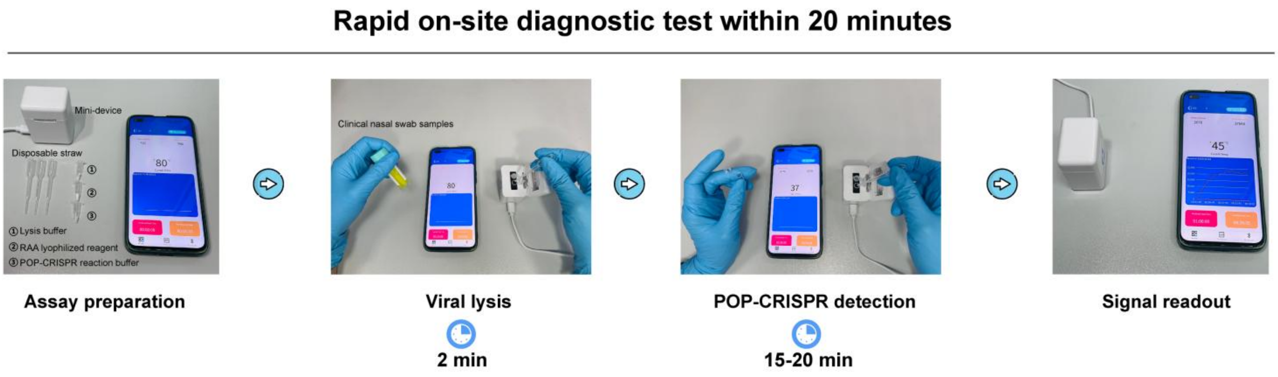

Supplementary Figure 24 | The operation workflow of on-site MP detection by POP-CRISPR using mini-device. This figure illustrates the stepwise procedure for performing on-site Mycoplasma pneumoniae (MP) detection with the portable POP-CRISPR mini-device. The workflow includes assay preparation, viral lysis, regent loading, and fluorescence readout, enabling rapid field-deployable molecular detection.

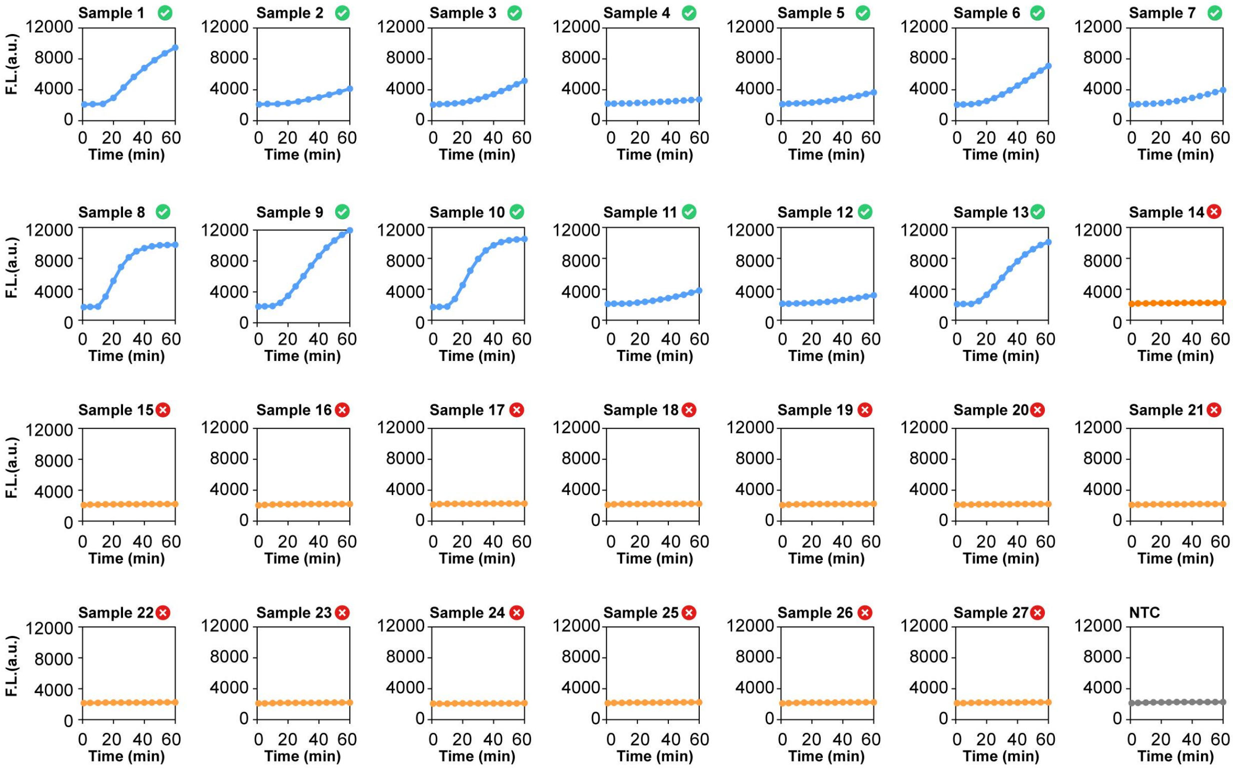

Supplementary Figure 25 | Real-time fluorescence curves obtained from POP-CRISPR experiments performed on Bio-Rad CFX96 Touch Real-Time PCR System. The experimental procedure was as follows: 10 min at 37°C followed by 50 min at 45°C, and the fluorescence signal was collected every minute throughout the whole process.

Supplementary Table 1 Nucleic acid sequences used in **Figure 1.**

Name	*Sequence (5'-3')*
PCR-F (294 bp)	GGGGCTGATAGTATTTA**NNNN**GGGGTTTGAGGTCCATT
PCR-R (294 bp)	CCAGGAGGTATCGGTGGAG
crRNA	UAAUUUCUACUAAGUGUAGAUGGGGUUUGAGGUCCAUUACA
FAM-6C-BHQ1	**FAM-**CCCCCC**-BHQ1**

Supplementary Table 2 Nucleic acid sequences used in **Figure 2 and Figure 3.**

Name	*Sequence (5'-3')*
PCR-F (1000 bp)	ATGTTTGTTTTTCTTGTTTTATTGCCACTAGTC
PCR-R (1000 bp)	TTGTAATATTAGGAAATCTAACAATAGATTCTGTTG
TTTA-crRNA1	UAAUUUCUACUAAGUGUAGAUUUGCCACUAGUCUCUAGUCA
TTTA-crRNA2	UAAUUUCUACUAAGUGUAGAUCAUUCAACUCAGGACUUGUU
TTTA-crRNA3	UAAUUUCUACUAAGUGUAGAUAUGAUGGUGUUUAUUUUGCU
TTTA-crRNA4	UAAUUUCUACUAAGUGUAGAUGAUUCGAAGACCCAGUCCCU
TTTA-crRNA5	UAAUUUCUACUAAGUGUAGAUAGAAUAUUGAUGGUUAUUUU
TTTA-crRNA6	UAAUUUCUACUAAGUGUAGAUCUUGCUUUACAUAGAAGUUA
TTTA-crRNA7	UAAUUUCUACUAAGUGUAGAUUUACCCUGACAAAGUUUUCA
TTTA-crRNA8	UAAUUUCUACUAAGUGUAGAUUUUUGCUUCCACUGAGAAGU
TTTA-crRNA9	UAAUUUCUACUAAGUGUAGAUUUACCACAAAAACAACAAAA
TTTA-crRNA10	UAAUUUCUACUAAGUGUAGAUUUCUAGUGCGAAUAAUUGCA
TTTA-crRNA11	UAAUUUCUACUAAGUGUAGAUAAAUAUAUUCUAAGCACACG
TTTA-crRNA12	UAAUUUCUACUAAGUGUAGAUGUGCGUGAUCUCCCUCAGGG
TTTA-crRNA13	UAAUUUCUACUAAGUGUAGAUGAACCAUUGGUAGAUUUGCC
TTTA-crRNA14	UAAUUUCUACUAAGUGUAGAUUUGCCACUAGUCUCUAGUCA
TTTA-crRNA15	UAAUUUCUACUAAGUGUAGAUGAGUCCAACCAACAGAAUCU
CCCC-crRNA16	UAAUUUCUACUAAGUGUAGAUCUGCAUACACUAAUUCUUUC
CCTC-crRNA17	UAAUUUCUACUAAGUGUAGAUAGUUUUACAUUCAACUCAGG
TTCC-crRNA18	UAAUUUCUACUAAGUGUAGAUAAUGUUACUUGGUUCCAUGC
TCTG-crRNA19	UAAUUUCUACUAAGUGUAGAUGGACCAAUGGUACUAAGAGG
TCCA-crRNA20	UAAUUUCUACUAAGUGUAGAUCUGAGAAGUCUAACAUAAUA
TTCA-crRNA21	UAAUUUCUACUAAGUGUAGAUAUUUUGUAAUGAUCCAUUUU
TTGG-crRNA22	UAAUUUCUACUAAGUGUAGAUUAGAUUUGCCAAUAGGUAUU
TCAC-crRNA23	UAAUUUCUACUAAGUGUAGAUUAGGUUUCAAACUUUACUUG
CTCA-crRNA24	UAAUUUCUACUAAGUGUAGAUGAAACAAAGUGUACGUUGAA
TTCA-crRNA25	UAAUUUCUACUAAGUGUAGAUAAAAUCUUAGGGAAUUUGUG
CCTC-crRNA26	UAAUUUCUACUAAGUGUAGAUUCUCAGAAACAAAGUGUACG
TCCA-crRNA27	UAAUUUCUACUAAGUGUAGAUACCAACAGAAUCUAUUGUUA
TCTG-crRNA28	UAAUUUCUACUAAGUGUAGAUAGAGAGGGUCAAGUGCACAG
TTCC-crRNA29	UAAUUUCUACUAAGUGUAGAUACUGAGAAGUCUAACAUAAU
TTCC-crRNA30	UAAUUUCUACUAAGUGUAGAUUUCAAGGUCCAUAAGAAAAG
TCAC-crRNA31	UAAUUUCUACUAAGUGUAGAUUGUAGAAAAAGGAAUCUAUC

TTCA-crRNA32	UAAUUUCUACUAAGUGUAGAUGGUUGGACAGCUGGUGCUGC
TTCA-crRNA33	UAAUUUCUACUAAGUGUAGAUGAGUUUAUUCUAGUGCGAAU
CTCA-crRNA34	UAAUUUCUACUAAGUGUAGAUGAAACAAAGUGUACGUUGAA
TTGG-crRNA35	UAAUUUCUACUAAGUGUAGAUACAGCUGGUGCUGCAGCUUA
CTCA-crRNA36	UAAUUUCUACUAAGUGUAGAUGUGGAAGCAAAAUAAACACC
TCCA-crRNA37	UAAUUUCUACUAAGUGUAGAUUAAGAAAAGGCUGAGAGACA
TCCA-crRNA38	UAAUUUCUACUAAGUGUAGAUGCCUCUUAUUAUGUUAGACU
TCAC-crRNA39	UAAUUUCUACUAAGUGUAGAUCAGGAGUCAAAUAACUUCUA

Supplementary Table 3 Nucleic acid sequences used in **Figure 4.**

Name	*Sequence (5'-3')*
HPV-16 RAA-F	GACTACTTGCAGTTGGACATCCCTATTTTCCT
HPV-16 RAA-R	CTACCTCAACACCTACACAGGCCCAAACCA
HPV-16 TTTA-crRNA	UAAUUUCUACUAAGUGUAGAUGAAUACAUUUACCUGACCCC
HPV-16 CCCC-crRNA	UAAUUUCUACUAAGUGUAGAUAAUAAGUUUGGUUUUCCUGA
HPV-18 RAA-F	TTGGAAGATGGTGATATGGTAGATACTGG
HPV-18 RAA-R	TATAAGGATTGAGGCACAGTGTCACCCAT
HPV-18 TTTA-crRNA	UAAUUUCUACUAAGUGUAGAUGUACAUUGCAAGAUACUAAA
HPV-18 CCTG-crRNA	UAAUUUCUACUAAGUGUAGAUAUUAUUUACAAAUGUCUGCA
N gene RAA-F	TTCCCTCGAGGACAAGGCGTTCCAATTAA
N gene RAA-R	TTCAAGGCTCCCTCAGTTGCAACCCATATGAT
N gene RT primer	ATGTGATCTTTTGGTGTA
N gene CCCA-crRNA	UAAUUUCUACUAAGUGUAGAUGUUCCUAGGUAGUAGAAAUA
crRNA-PM	UAAUUUCUACUAAGUGUAGAUCCUGCUGUUUGGAUAUUGUG
crRNA-M1-2	UAAUUUCUACUAAGUGUAGAUGGUGCUGUUUGGAUAUUGUG
crRNA-M3-4	UAAUUUCUACUAAGUGUAGAUCCGACUGUUUGGAUAUUGUG
crRNA-M5-6	UAAUUUCUACUAAGUGUAGAUCCUGAGGUUUGGAUAUUGUG
crRNA-M7-8	UAAUUUCUACUAAGUGUAGAUCCUGCUAAUUGGAUAUUGUG
crRNA-M9-10	UAAUUUCUACUAAGUGUAGAUCCUGCUGUACGGAUAUUGUG
crRNA-M11-12	UAAUUUCUACUAAGUGUAGAUCCUGCUGUUUCCAUAUUGUG
crRNA-M13-14	UAAUUUCUACUAAGUGUAGAUCCUGCUGUUUGGUCAUUGUG
crRNA-M15-16	UAAUUUCUACUAAGUGUAGAUCCUGCUGUUUGGAUGAUGUG
crRNA-M17-18	UAAUUUCUACUAAGUGUAGAUCCUGCUGUUUGGAUAUCAUG
crRNA-M19-20	UAAUUUCUACUAAGUGUAGAUCCUGCUGUUUGGAUAUUGGA
crRNA-M3	UAAUUUCUACUAAGUGUAGAUCCAGCUGUUUGGAUAUUGUG
crRNA-M4	UAAUUUCUACUAAGUGUAGAUCCUCCUGUUUGGAUAUUGUG
crRNA-M9	UAAUUUCUACUAAGUGUAGAUCCUGCUGUAUGGAUAUUGUG
crRNA-M10	UAAUUUCUACUAAGUGUAGAUCCUGCUGUUAGGAUAUUGUG
crRNA-M11	UAAUUUCUACUAAGUGUAGAUCCUGCUGUUUCGAUAUUGUG
crRNA-M12	UAAUUUCUACUAAGUGUAGAUCCUGCUGUUUGCAUAUUGUG
crRNA-M13	UAAUUUCUACUAAGUGUAGAUCCUGCUGUUUGGUUAUUGUG
crRNA-M14	UAAUUUCUACUAAGUGUAGAUCCUGCUGUUUGGAAAUUGUG
crRNA-M15	UAAUUUCUACUAAGUGUAGAUCCUGCUGUUUGGAUUUUGUG
crRNA-M16	UAAUUUCUACUAAGUGUAGAUCCUGCUGUUUGGAUAAUGUG

Supplementary Table 4 Nucleic acid sequences used in **Figure 5 and Figure 6.**

Name	*Sequence (5'-3')*
M.P.(P1) RAA-F	CTCACCGTAGTGGGACACTTCACAAGTACC
M.P.(P1)RAA-R	TCGGGTGGGATCATACGTGGTTTGTTGACT
M.P.(P1) TTTA-crRNA	UAAUUUCUACUAAGUGUAGAUGCUACACCCGCCCUGACGAG
M.P.(P1)TTAG-crRNA	UAAUUUCUACUAAGUGUAGAUCCCUGACGAGGUCGCGCUGC
M.P.(23S rRNA) RAA-F	TTGGAAGATGGTGATATGGTAGATACTGG
M.P.(23S rRNA)RAA-R	TATAAGGATTGAGGCACAGTGTCACCCAT
M.P.(23S rRNA)-GTTA-crRNA-wt	UAAUUUCUACUAAGUGUAGAUGGCGCAACGGGACGGAAAGA
M.P.(23S rRNA)-GTTA-crRNA-mut	UAAUUUCUACUAAGUGUAGAUGGCGCAACGGGACGGGAAGA

Supplementary Table 5 Plasmid sequences used in this study (ASFV).

Name	*Sequence (5’-3’)*
Plasmid sequence used in Fig.1 (ASFV)	AAGTCCAGGAAATTCATTCACCAAATCCTTTTGCGATGCAAGCTTTA<br>TGGTGATAAAGCGCTCGCCGAAGGGAATGGATACTGAGGGAATAGC<br>AAGGTTCACGTTCTCGTTAAACCAAAAGCGCAGCTTAATCCAGAGC<br>GCAAGAGGGGGCTGATAGTATTTAGGGGTTTGAGGTCCATTACAGCT<br>GTAATGAACATTACGTCTTATGTCCAGATACGTTGCGTCCGTGATAG<br>GAGTGATATCTTGTTTACCTGCTGTTTGGATATTGTGAGAGTTCTCGG<br>GAAAATGTTGTGAAAGAAATTTCGGGTTGGTATGGCTGCACGTTCG<br>CTGCGTATCATTTTCATCGGTAAGAATAGGTTTGCTTTGGTGCGGCTT<br>GTGCAAATCATGAATGTTGCATAGGAGAGGGCCACTGGTTCCCTCC<br>ACCGATACCTCCTGGCCGACCAAGTGCTTATATCC

Supplementary Table 6 Plasmid sequences used in this study (SARS-CoV-2 S-gene).

Name	*Sequence (5'-3')*
Plasmid sequence used in Fig.2 (SARS-CoV-2 S-gene)	ATGTTTGTTTTTCTTGTTTTATTGCCACTAGTCTCTAGTCAGTGTGTT<br>AATCTTACAACCAGAACTCAATTACCCCCTGCATACACTAATTCTTTC<br>ACACGTGGTGTTTATTACCCTGACAAAGTTTTCAGATCCTCAGTTTT<br>ACATTCAACTCAGGACTTGTTCTTACCTTTCTTTTCCAATGTTACTTG<br>GTTCCATGCTATACATGTCTCTGGGACCAATGGTACTAAGAGGTTTG<br>ATAACCCTGTCCTACCATTTAATGATGGTGTTTATTTTGCTTCCACTG<br>AGAAGTCTAACATAATAAGAGGCTGGATTTTTGGTACTACTTTAGAT<br>TCGAAGACCCAGTCCCTACTTATTGTTAATAACGCTACTAATGTTGTT<br>ATTAAAGTCTGTGAATTTCAATTTTGTAATGATCCATTTTTGGGTGTT<br>TATTACCACAAAAACAACAAAAGTTGGATGGAAAGTGAGTTCAGAG<br>TTTATTCTAGTGCGAATAATTGCACTTTTGAATATGTCTCTCAGCCTT<br>TTCTTATGGACCTTGAAGGAAAACAGGGTAATTTCAAAAATCTTAGG<br>GAATTTGTGTTTAAGAATATTGATGGTTATTTTAAAATATATTCTAAGC<br>ACACGCCTATTAATTTAGTGCGTGATCTCCCTCAGGGTTTTTCGGCTT<br>TAGAACCATTGGTAGATTTGCCAATAGGTATTAACATCACTAGGTTTC<br>AAACTTTACTTGCTTTACATAGAAGTTATTTGACTCCTGGTGATTCTT<br>CTTCAGGTTGGACAGCTGGTGCTGCAGCTTATTATGTGGGTTATCTT<br>CAACCTAGGACTTTTCTATTAAAATATAATGAAAATGGAACCATTAC<br>AGATGCTGTAGACTGTGCACTTGACCCTCTCTCAGAAACAAAGTGT<br>ACGTTGAAATCCTTCACTGTAGAAAAAGGAATCTATCAAACTTCTAA<br>CTTTAGAGTCCAACCAACAGAATCTATTGTTAGATTTCCTAATATTAC<br>AA

Supplementary Table 7 Plasmid sequences used in this study (SARS-CoV-2 N-gene).

Name	*Sequence (5'-3')*
Plasmid sequence used in Fig.4 (SARS-CoV-2 N-gene)	GTTTACCCAATAATACTGCGTCTTGGTTCACCGCTCTCACTCAACAT GGCAAGGAAGACCTTAAATTCCCTCGAGGACAAGGCGTTCCAATTA ACACCAATAGCAGTCCAGATGACCAAATTGGCTACTACCGAAGAGC TACCAGACGAATTCGTGGTGGTGACGGTAAAATGAAAGATCTCAGT CCAAGATGGTATTTCTACTACCTAGGAACTGGGCCAGAAGCTGGAC TTCCCTATGGTGCTAACAAAGACGGCATCATATGGGTTGCAACTGAG GGAGCCTTGAATACACCAAAAGATCACATTGGCACCCGCAATCCTG CTAACAATGCTGCAATCGTGCTACAACTTCCTCAAGGAACAACATTG CCAAAAGGCTTCTACGCAGAAGGGAGCAG

Supplementary Table 8 The *Ct* values of 33 clinical HPV-16 samples measured by qRT-PCR.

**Sample ID**	**1**	**2**	**3**	**4**	**5**	**6**	**7**	**8**	**9**
Hpv16 (Ct)	21.74	27.668	28.72	35.66	33.95	11.91	19.24	36.74	30.52
**Sample ID**	**10**	**11**	**12**	**13**	**14**	**15**	**16**	**17**	**18**
Hpv16 (Ct)	21.86	28.91	24.25	14.53	32.42	31.92	26.86	31.59	24.17
**Sample ID**	**19**	**20**	**21**	**22**	**23**	**24**	**25**	**26**	**27**
Hpv16 (Ct)	26.56	31.87	23.87	27.95	16.51	27.98	25.72	28.97	39.883
**Sample ID**	**28**	**29**	**30**	**31**	**32**	**33**			
Hpv16 (Ct)	-	-	-	-	-	-			

Supplementary Table 9 The *Ct* values of 67 clinical MP samples measured by qRT-PCR.

**Sample ID**	**1**	**2**	**3**	**4**	**5**	**6**	**7**	**8**	**9**
MP (Ct)	34.91	28.77	33.79	27.37	36.23	26.59	36.09	34.91	32.67
**Sample ID**	**10**	**11**	**12**	**13**	**14**	**15**	**16**	**17**	**18**
MP (Ct)	28.40	33.96	33.99	26.28	30.61	30.03	30.83	32.98	23.48
**Sample ID**	**19**	**20**	**21**	**22**	**23**	**24**	**25**	**26**	**27**
MP (Ct)	29.46	28.69	35.06	26.75	32.24	30.35	29.13	30.42	34.03
**Sample ID**	**28**	**29**	**30**	**31**	**32**	**33**	**34**	**35**	**36**
MP (Ct)	27.89	32.69	23.68	28.68	30.49	26.17	27.88	34.19	30.54
**Sample ID**	**37**	**38**	**39**	**40**	**41**	**42**	**43**	**44**	**45**
MP (Ct)	32.62	32.06	25.48	27.94	30.74	33.57	25.87	31.82	35.23
**Sample ID**	**46**	**47**	**48**	**49**	**50**	**51**	**52**	**53**	**54**
MP (Ct)	28.43	28.44	30.25	30.45	37.60	-	-	-	-
**Sample ID**	**55**	**56**	**57**	**58**	**59**	**60**	**61**	**62**	**63**
MP (Ct)	-	-	-	-	-	-	-	-	-
**Sample ID**	**64**	**65**							
MP (Ct)	-	-							

Supplementary Table 10 The *Ct* values of 11 clinical MP samples for macrolide-resistant mutation detection measured by qRT-PCR.

**Sample ID**	**1**	**2**	**3**	**4**	**5**	**6**	**7**	**8**	**9**
FAM (Ct)	29.91	28.75	30.32	32.01	24.62	26.88	26.7	27.18	36.76
VIC (Ct)	27.28	25.21	27.59	29.44	21.94	23.99	23.51	24.16	34.05
**Sample ID**	**10**	**11**							
FAM (Ct)	UNDET	UNDET							
VIC (Ct)	28.75	23.83							

A2063G mutation: FAM Ct<35.33, VIC<35.01; WT: FAM Ct⩾35.33 or UNDET, VIC<35.01

Supplementary Table 11 The *Ct* values of 27 clinical MP samples used in Fig.6 c-d measured by qRT-PCR.

**Sample ID**	**1**	**2**	**3**	**4**	**5**	**6**	**7**	**8**	**9**
MP (Ct)	24.44	32.48	33.79	36.01	34.93	31.16	25.71	22.41	23.82
**Sample ID**	**10**	**11**	**12**	**13**	**14**	**15**	**16**	**17**	**18**
MP (Ct)	24.77	32.87	33.97	21.99	-	-	-	-	-
**Sample ID**	**19**	**20**	**21**	**22**	**23**	**24**	**25**	**26**	**27**
MP (Ct)	-	-	-	-	-	-	-	-	-

Supplementary Table 12 Comparison between POP-CRISPR and qPCR.

**Parameter**	**POP-CRISPR**	**qPCR**
Reaction mode	One-pot (RAA + Cas12a)	One-pot (thermal cycling)
Temperature	37°C (amplification) → 45–55°C (CRISPR detection)	Cyclic heating
Time to result	Within 20 min	60-90 min
Analytical LOD	0.5aM (≈4-5 copies)	1-10 copies
Clinical sample sensitivity	HPV-16 Ct=35.7<br>MP Ct=36.2	Ct≤37 considered positive
Concordance	100% with qPCR	Gold standard
Mismatch discrimination	Single-base resolution (≥53°C, non-canonical PAMs	Limited
Sample preparation	Rapid 2-min heat lysis for nucleic acid release	Standardized nucleic acid extraction required (>20 min)
Instrumentation	Portable isothermal fluorescence detector	Thermal cycler (bench-top)

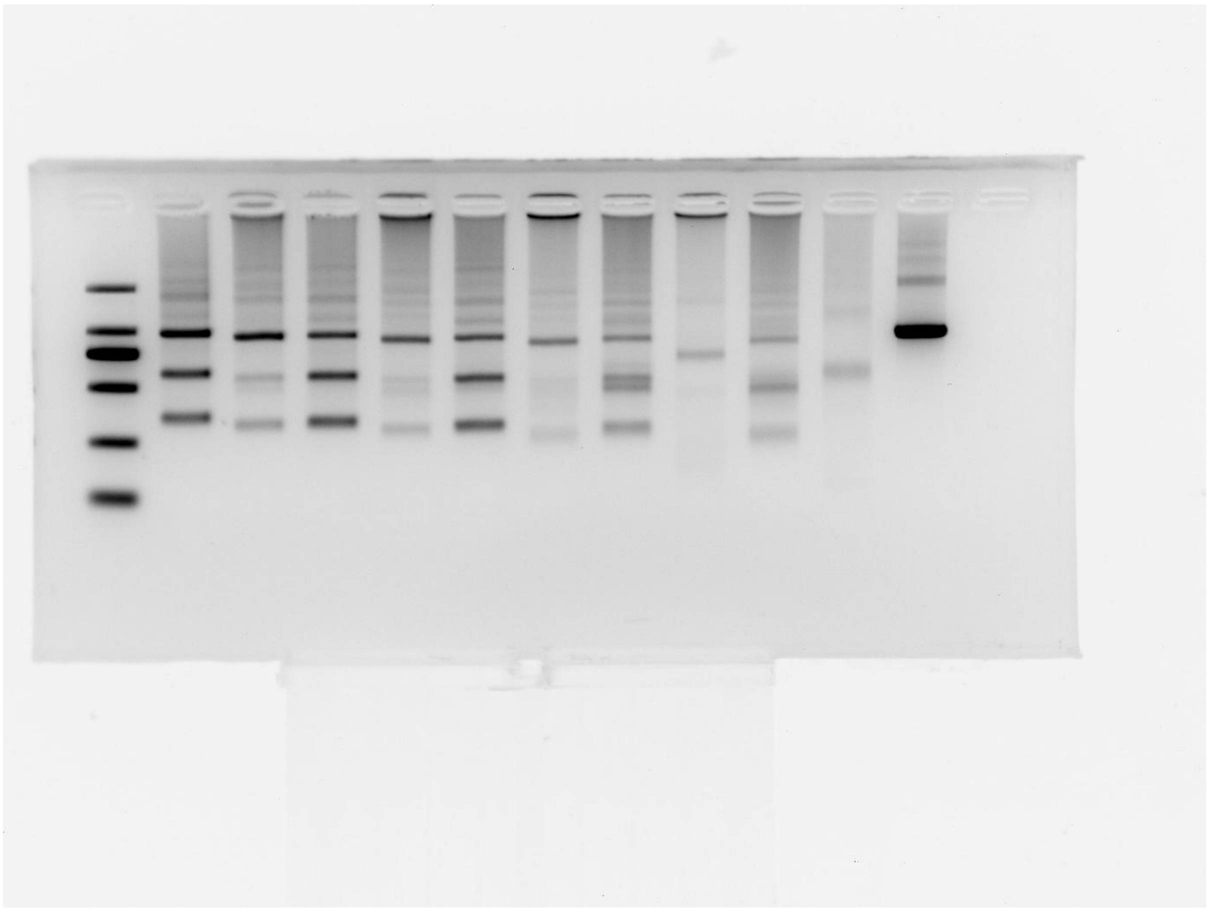

Uncropped scans of agrose gel in Supplementary Fig.14a （37°C and 45°C）

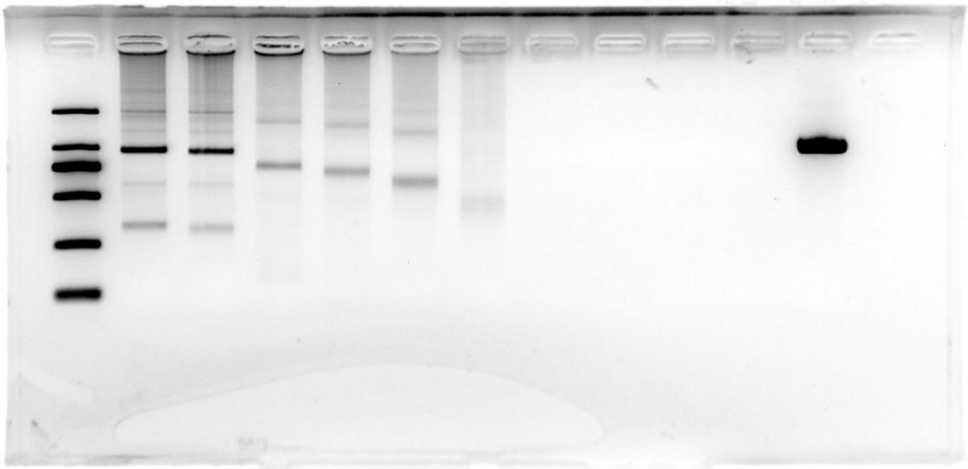

Uncropped scans of agrose gel in Supplementary Fig.14a（55°C and 53°C）

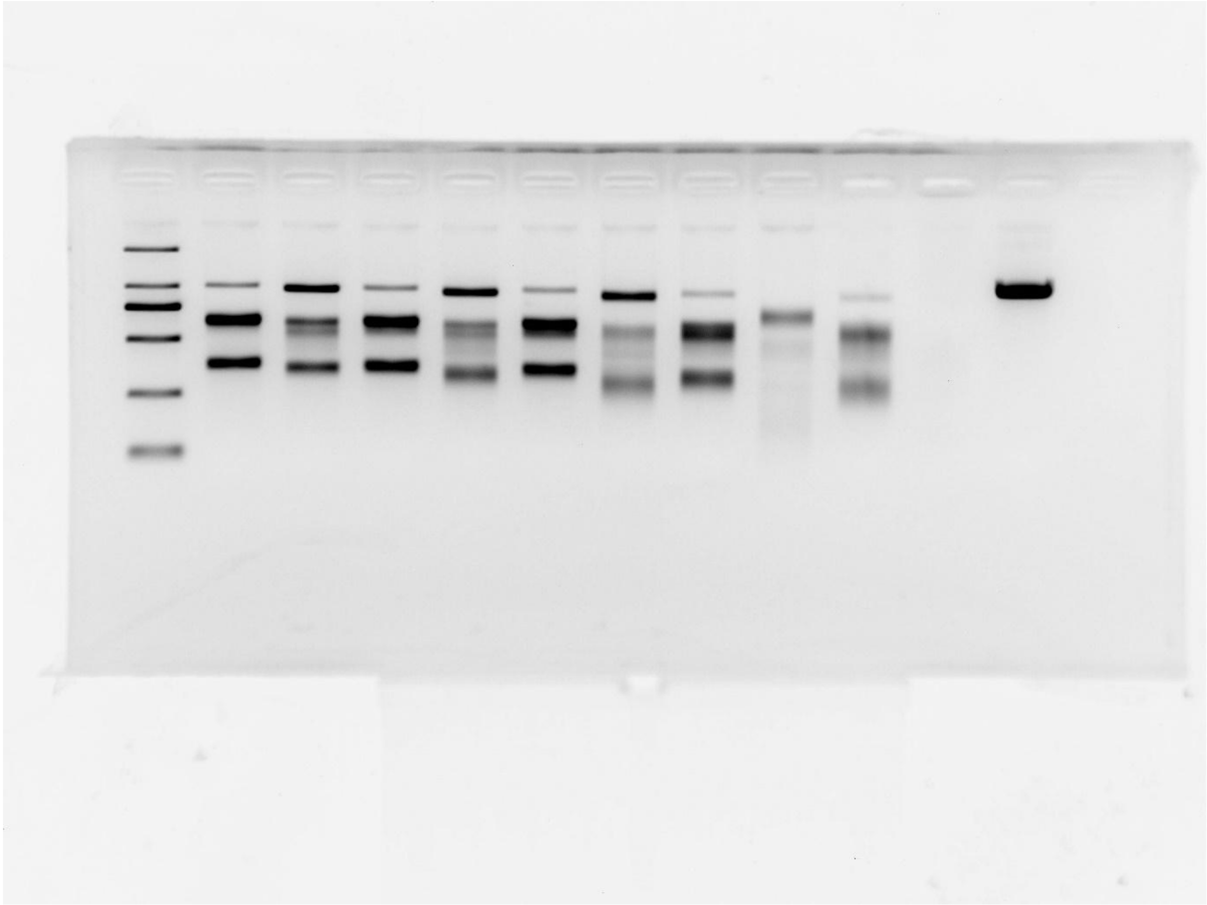

Uncropped scans of agrose gel in Supplementary Fig.14b（37°C and 45°C）

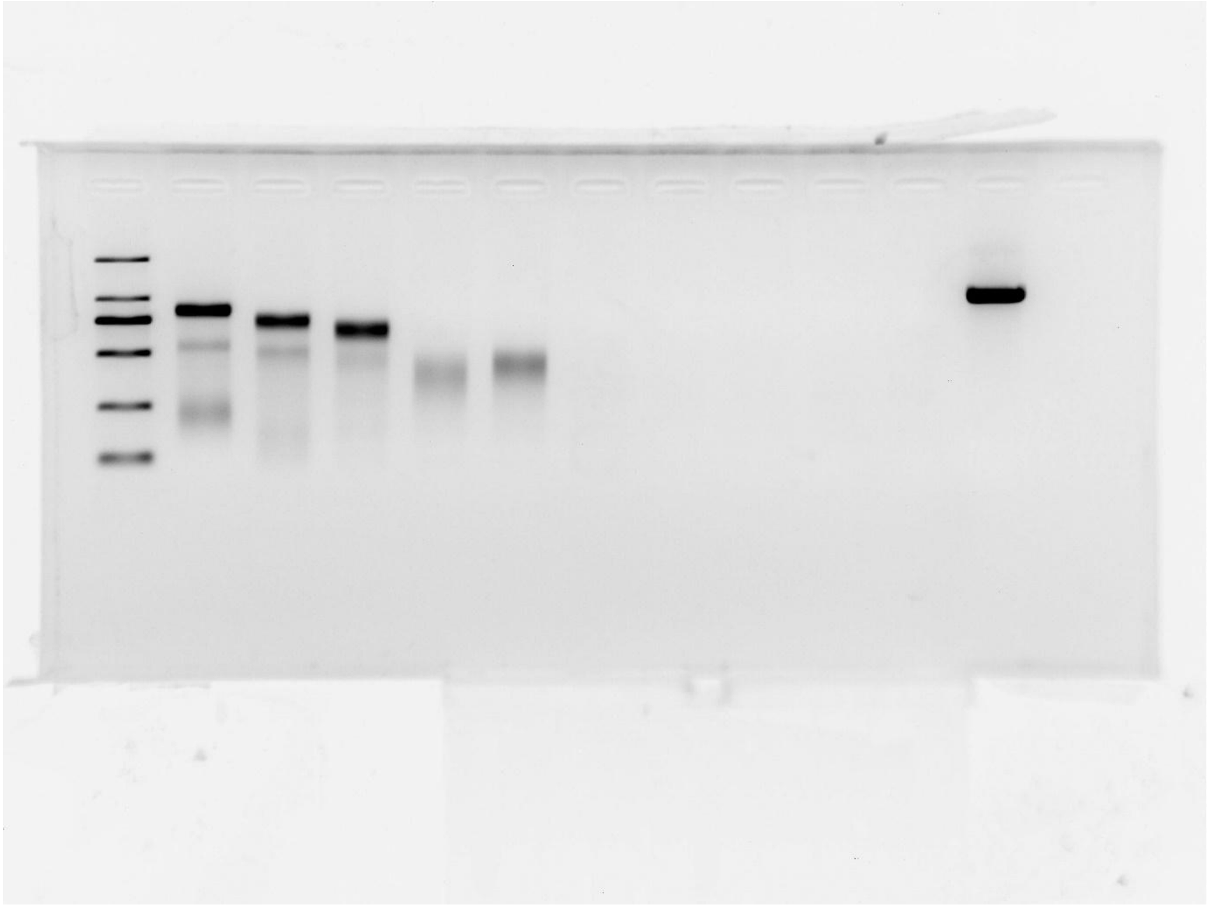

Uncropped scans of agrose gel in Supplementary Fig.14b（53°C and 55°C）

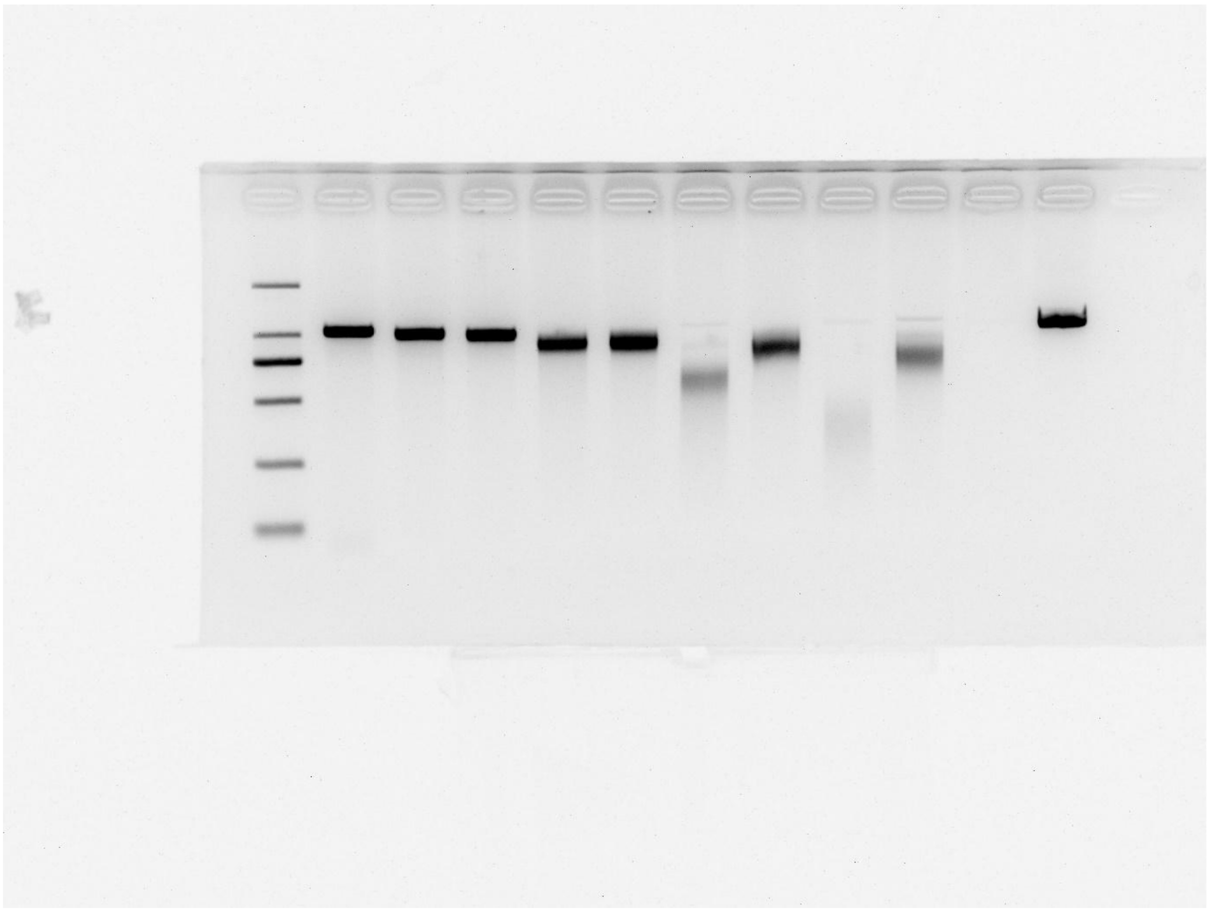

Uncropped scans of agrose gel in Supplementary Fig.15b（37°C and 45°C）

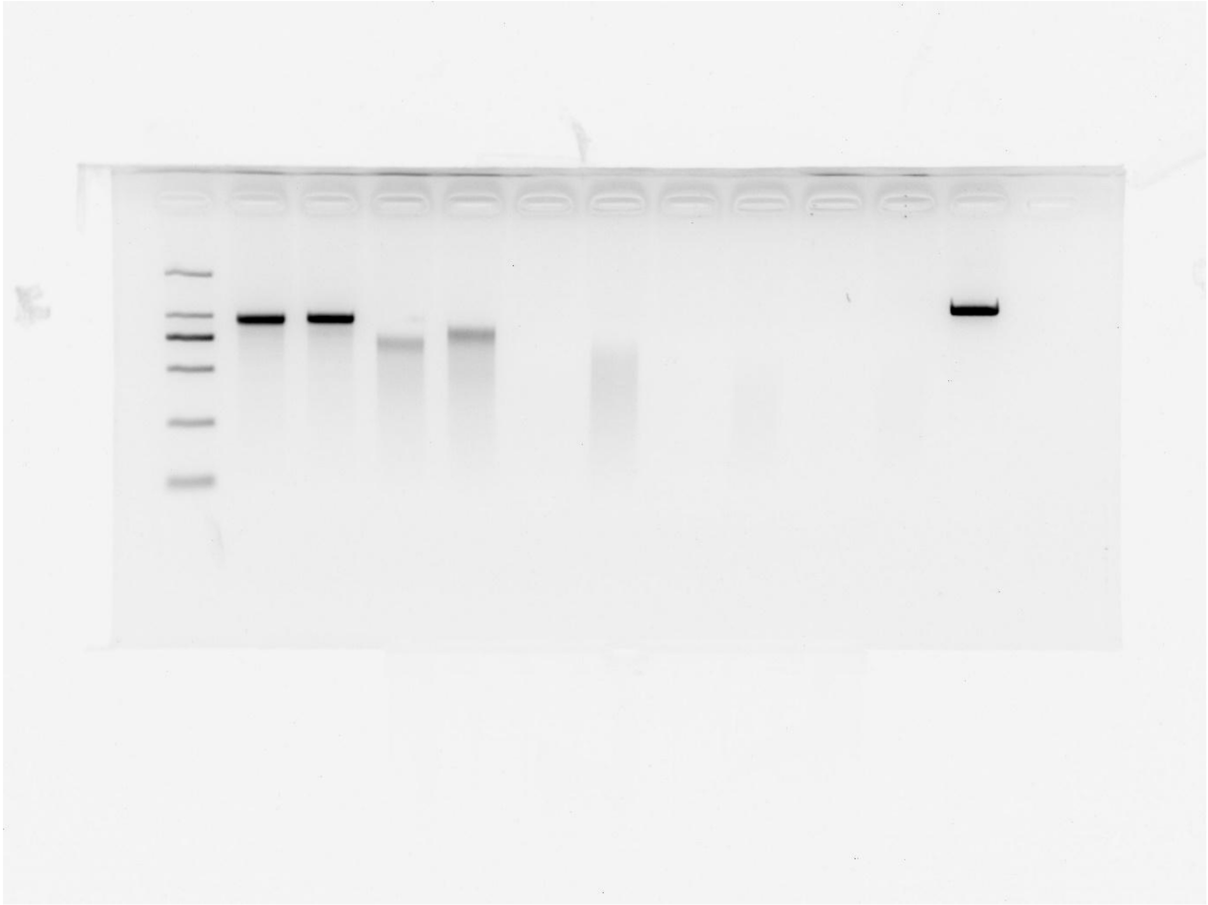

Uncropped scans of agrose gel in Supplementary Fig.15b（53°C and 55°C）
