# Supplementary material for: Identification of thermotolerant non-canonical PAMs for robust one-pot CRISPR-Cas12a detection
Source: Nat Commun. 2026 Jan 16;17:1771. doi: 10.1038/s41467-026-68476-3 (PMC12917250; doi:10.1038/s41467-026-68476-3)
Supplement: Supplementary file 2 — Description of Additional Supplementary Files [file 41467_2026_68476_MOESM2_ESM.pdf]

Description of Additional Supplementary Files

File Name: Supplementary Data 1
Description: Real-time fluorescence kinetic curves showing the trans-cleavage activities of 256 PAM sites at reaction temperatures of 37 °C and 45 °C. Fluorescence intensity was monitored continuously to assess the Cas12a trans-cleavage activity for each of the 256 PAM targets. Each curve represents the kinetic profile of ssDNA reporter cleavage over time.
